# The Efficacy of Virtual Reality on the Rehabilitation of Musculoskeletal Diseases: Umbrella Review

**DOI:** 10.2196/64576

**Published:** 2025-04-25

**Authors:** Peiyuan Tang, Yangbin Cao, Djandan Tadum Arthur Vithran Vithran, Wenfeng Xiao, Ting Wen, Shuguang Liu, Yusheng Li

**Affiliations:** 1 Department of Orthopedics, Xiangya Hospital Central South University Changsha China; 2 National Clinical Research Center for Geriatric Disorders, Xiangya Hospital Central South University Changsha China; 3 Xiangya School of Medicine Central South University Changsha China; 4 Department of Joint Surgery, Honghui Hospital Xi'an Jiaotong University Xi'an China

**Keywords:** virtual reality, VR, umbrella review, musculoskeletal diseases, orthopedics, efficacy

## Abstract

**Background:**

Musculoskeletal disorders cause pain, disability, and financial burdens, with rising prevalence. Virtual reality (VR) offers immersive, digital rehabilitation potential, improving motor functions and pain management.

**Objective:**

To evaluate the impact of VR on the rehabilitation of musculoskeletal disorders and to assess the consistency of evidence provided by existing systematic reviews and meta-analyses, this study focuses on musculoskeletal disorders, which encompass conditions affecting the locomotor system, such as arthritis, joint deformities, and injuries. VR therapy leverages immersive digital environments to enhance rehabilitation through digital exercises and simulations.

**Methods:**

The PubMed or MEDLINE, Embase, and Cochrane Library databases were systematically searched for relevant papers published up to April 2024. Literature screening, quality assessment, and data extraction were conducted according to predefined inclusion and exclusion criteria. The methodological quality of the included meta-analyses was evaluated using the Measurement Tool to Assess Systematic Reviews 2. The Grading of Recommendations Assessment, Development, and Evaluation system was used to classify the evidence level for each outcome as high, moderate, low, or very low. Additionally, the evidence was categorized into 5 levels based on classification criteria: I (convincing), II (highly suggestive), III (suggestive), IV (weak), and nonsignificant.

**Results:**

This umbrella review synthesized data from 14 meta-analyses published between 2019 and 2024, involving a total of 13,184 patients. In total, 7 meta-analyses received high Measurement Tool to Assess Systematic Reviews 2 ratings, 7 were rated moderate, and the remainder were rated low. VR demonstrated promising results in musculoskeletal rehabilitation, significantly reducing knee pain (mean difference [MD] –1.38, 95% CI –2.32 to –0.44; *P*=.004; *I*^2^=94%) and improving balance. For patients with fibromyalgia syndrome, VR effectively reduced pain (standardized mean difference [SMD] –0.45, 95% CI –0.70 to –0.20; *P*<.001), fatigue (SMD –0.58, 95% CI –1.01 to –0.14; *P*=.01), anxiety (SMD –0.50, 95% CI –0.908 to –0.029; *P*=.04), and depression (SMD –0.62, 95% CI –0.76 to –0.15; *P*=.003) while also enhancing quality of life. In individuals with back pain, VR alleviated pain-related fears (MD –5.46, 95% CI –9.40 to –1.52; *P*=.007; *I*^2^=90%) and reduced pain intensity (MD –1.43, 95% CI –1.86 to –1.00; *P*<.001; *I*^2^=95%). After arthroplasty, VR improved knee functionality (MD 8.30, 95% CI 6.92-9.67; *P*<.001; *I*^2^=24%) and decreased anxiety (MD –3.95, 95% CI –7.76 to –0.13; *P*=.04; *I*^2^=0%).

**Conclusions:**

VR demonstrates significant potential in the rehabilitation of various musculoskeletal conditions. It effectively alleviates pain, enhances psychological well-being, and facilitates the recovery of motor function in patients.

**Trial Registration:**

PROSPERO CRD42024538469; https://www.crd.york.ac.uk/PROSPERO/view/CRD42024538469

## Introduction

Musculoskeletal disorders encompass a range of conditions affecting the human locomotor system, including arthritis, joint deformities, and various types of injuries [1]. These disorders are a leading cause of disability. The adverse effects of musculoskeletal disorders manifest in several ways, including persistent pain, reduced quality of life, and declines in physical function and mobility [2]. Managing these diseases is challenging, requiring substantial medical resources and long-term treatment, thereby posing a financial burden [3]. As the population ages, the prevalence of these conditions is expected to increase [4].

In recent years, virtual reality (VR) technology has gained recognition as a promising tool in medicine and rehabilitation [5]. VR substitutes the surrounding environment with graphical and auditory effects, allowing users to interact with a virtual world [6]. Its digital, immersive, and multimodal feedback characteristics offer significant potential for motor rehabilitation [7]. In orthopedics, VR is an essential component of patient rehabilitation care, helping patients overcome pain and enhance motor abilities through simulated environments and gamified therapies [8]. This review of existing meta-analyses aims to evaluate the efficacy of VR interventions on musculoskeletal disorders and assess the consistency of evidence from these studies. By providing decision-makers with a comprehensive source of high-quality research, this umbrella review seeks to methodically integrate relevant outcomes to create an extensive evidence base.

## Methods

### Study Design

An umbrella review was conducted to assess and compile evidence from multiple meta-analyses [9,10]. We followed the protocols outlined in the Cochrane Handbook [10-12]. The review was registered on the PROSPERO website (CRD42024538469) and adhered to the PRIOR (Preferred Reporting Items for Overviews of Reviews) and the AMSTAR 2 (A Measurement Tool to Assess Systematic Reviews 2) guidelines (Multimedia Appendix 1) [13,14].

### Search Methodology

The Embase, PubMed or MEDLINE, and Cochrane Library databases were searched until April 2024 to identify relevant literature. Various topic terms and keywords were used during the literature retrieval process [15]. The search terms used in English included “virtual reality,” “musculoskeletal diseases,” “orthopedics,” “meta-analysis,” and others (Multimedia Appendix 2).

### Study Selection

The inclusion and exclusion criteria for participant selection are detailed in Textbox 1. For the literature search, the following filters were applied:

- Time range: From the inception of the database to April 2024.
- Study type: Only systematic reviews or meta-analyses were selected.
- Language restriction: Only studies published in English or specific other languages were included.

**Inclusion and exclusion criteria for this study.**

**Inclusion criteria**

- Systematic reviews that conducted meta-analyses.
- Peer-reviewed journal papers.
- Meta-analyses evaluating clinical outcomes, particularly treatment effects for musculoskeletal disorders.
- Studies published in English.
- Reviews containing empirical research data and statistical analysis.

**Exclusion criteria**

- Studies not related to virtual reality interventions or musculoskeletal disorders.
- Non–peer-reviewed papers or literature (eg, conference abstracts, patents, and gray literature).
- Studies not related to virtual reality interventions or musculoskeletal disorders.
- Systematic reviews that do not include a meta-analysis.
- Descriptive reviews or theoretical papers without empirical research data or statistical analysis.

### Overlapping Discovery and Processing

If 2 or more meta-analyses evaluate the same outcome, there is a possibility that the original studies they included may overlap. We use the Graphical Representation of Overlap for Overviews (GROOVE) to identify where original research overlaps [16]. GROOVE is a tool that visually represents this overlap. Using GROOVE, we can categorize overlap into 4 levels: very high (>15%), high (10% to <15%), moderate (5% to <10%), and slight (<5%). In this study, only a few original studies overlap among the extracted outcomes. The results of the GROOVE analysis are presented in Multimedia Appendix 3. When overlap occurs, the following measures are implemented: (1) if the overlap involves Cochrane reviews and non-Cochrane reviews, the results of the Cochrane reviews are prioritized. (2) If a high degree of overlap (≥10%) is found between 2 or more non-Cochrane reviews, the results of the meta-analysis with the highest AMSTAR 2 score are given priority. (3) If the AMSTAR 2 scores are consistent, the results of the meta-analysis that includes the highest number of randomized controlled trials (RCTs) or the most recent publication are prioritized.

### Obtaining Data and Evaluating Its Quality

Two authors (PT and TW) worked independently to retrieve data and assess its quality, while a third author (YL) was available to resolve any disagreements. The main results extracted for this study include the visual analog scale (VAS), the Western Ontario and McMaster Universities Osteoarthritis Index (WOMAC), range of motion (ROM), timed up and go test (TUG), and others. Pain levels in this study were represented by VAS scores. Additionally, the study extracted the sex ratio of the included studies, average age, number of included meta-analyses, and number of individuals. Two authors (PT and TW) independently assessed the methodological quality of the included meta-analyses using AMSTAR 2. When disputes arose, the senior author (YL) mediated discussions to reach a consensus [17]. Using the Grading of Recommendations Assessment, Development, and Evaluation (GRADE) approach [18], we appraised the level of evidence for each result, categorizing them as high, moderate, low, or very low. Furthermore, we sorted the evidence into 5 levels according to the criteria for evidence classification: I (convincing evidence), II (highly suggestive evidence), III (suggestive evidence), IV (weak evidence), and nonsignificant. Detailed criteria for the classification of evidence are shown in Textbox 2 [9].

**Evidence classification criteria.**

Class I: >1000 cases (or >20,000 participants for continuous outcomes), statistical significance at *P*<10^−6^ (random effects), no evidence of small study effects and excess significance bias, 95% prediction interval excluded null value, and no large heterogeneity (I^2^<50%)

Class II: >1000 cases (or >20,000 participants for continuous outcomes), statistical significance at *P*<10^−6^ (random effects), and largest study with 95% CI excluding null value

Class III: >1000 cases (or >20,000 participants for continuous outcomes) and statistical significance at *P*<.001

Class IV: Remaining significant associations with *P*<.05

Nonsignificant: *P*>.05

### Data Synthesis

The studies included in this research are meta-analyses, with results presented through descriptive analysis and tabular formats (Multimedia Appendix 4) [19-32]. Basic information from the included meta-analyses is provided in the study characteristics. The statistical measures used in this study include the mean difference (MD) and standardized mean difference (SMD). Heterogeneity among studies is measured using the *I*^2^ statistic, which ranges from 0% to 100%. This statistic reflects the proportion of overall variation attributable to heterogeneity. The interpretation of *I*^2^ values was as follows: low (*I*^2^<25%), low to moderate (*I*^2^=25%-50%), moderate to substantial (*I*^2^=50%-75%), or substantial (*I*^2^>75%).

## Results

### Search Results

**Figure 1:**
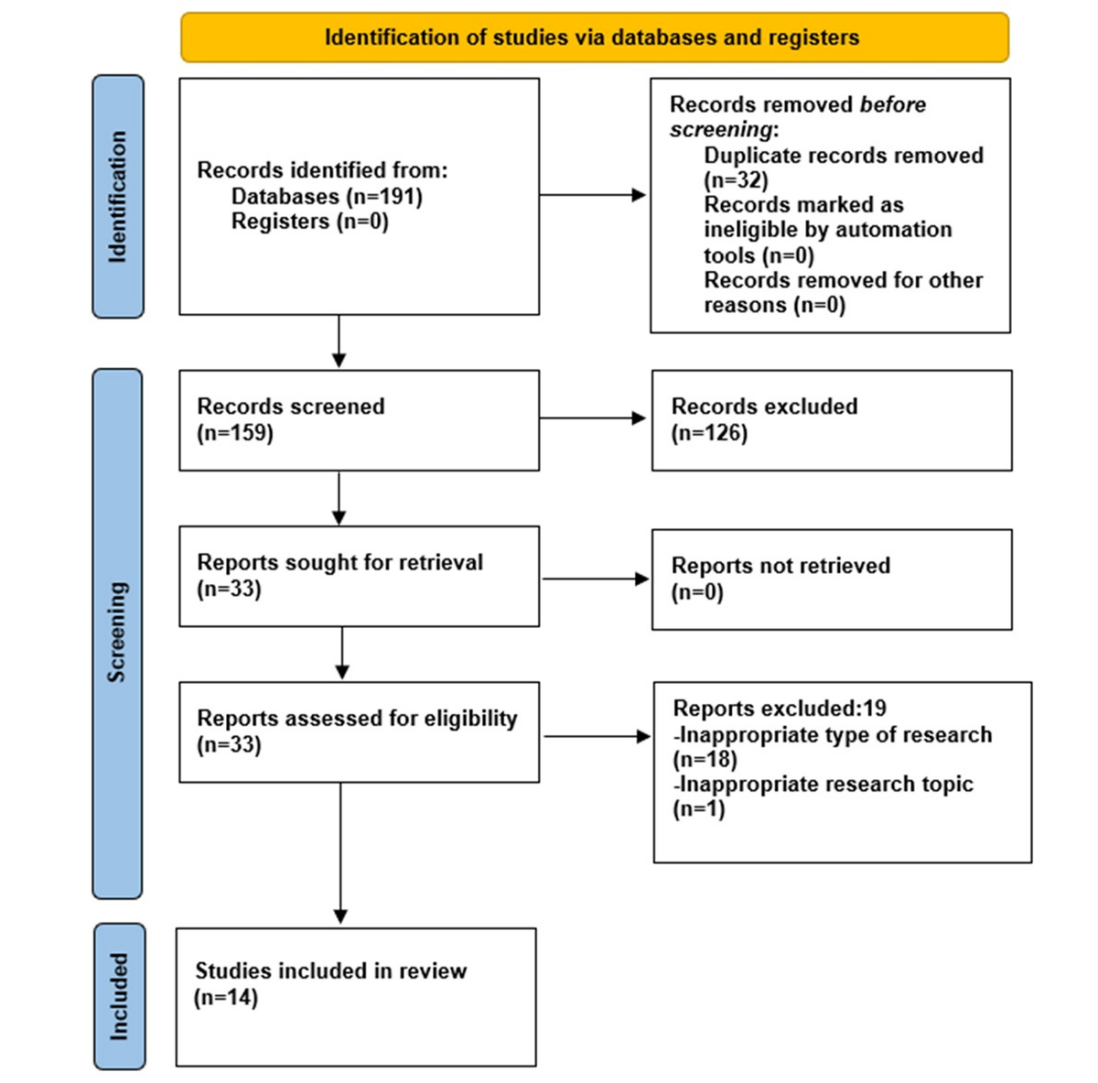
The PRISMA (Preferred Reporting Items for Systematic Reviews and Meta-Analyses) flow diagram to show study selection.

A total of 191 systematic reviews and meta-analyses were initially identified using the search strategy. After removing duplicates, 32 studies were eliminated. A further 126 studies were excluded by closely examining the titles and abstracts and applying the inclusion and exclusion criteria. Finally, after a full-text review, 18 studies were excluded (Multimedia Appendix 5), resulting in the inclusion of 14 studies. The process for screening the literature is illustrated in Figure 1.

### Study Characteristics

This umbrella review synthesized data from a total of 14 meta-analyses published between 2019 and 2024, involving 13,184 patients. Table 1 presents the basic characteristics of the included meta-analyses. The meta-analyses in this review addressed a range of common musculoskeletal disorders, including back pain, neck pain, knee joint pain, arthroplasty, fibromyalgia syndrome in the female population, and other musculoskeletal conditions. A total of 14 studies gave the average age of patients, and in 4 of these studies [19-31,33], the average age of patients was 60 years and older [19,26,28,31]. A total of 9 studies gave the sex ratio of included patients [19,20,22,23,25,27,28,30,31], of which 1 had a lower proportion of female than male patients [27]. A total of 14 studies gave the number of included patients [19-31,33], of which only 4 studies had more than 1000 patients [20,27,31,33]. In total, 7 studies analyzed the efficacy of immersive VR [21-23,25-27,29]. The remaining meta-analyses included both immersive and nonimmersive VR studies. Evidence evaluation was conducted on the extracted outcomes (Multimedia Appendix 4). A total of 22 outcomes were rated as level IV, 1 outcome was rated as level III, and 16 outcomes were rated as nonsignificant. Additionally, these outcomes were assessed using the GRADE approach: 4 outcomes were rated as moderate, 8 outcomes as very low, 26 outcomes as low, and 1 outcome as high. The main conclusions from all included studies in this review were also extracted (Multimedia Appendix 6) [19-32]. A total of 7 meta-analyses received a high AMSTAR 2 rating, 7 were rated as moderate, and the remaining meta-analyses were rated as low (Table 2).

**Table 1:** Basic information about study patients was included.

Study	Year	Region	Last search date	Condition	Age (years)	Sex ratio (female) (%)	Sample size	Studies included, n (%)
Su et al [19]	2024	China	February 2023	Total knee arthroplasty	68.18	64	989	14
Li et al [20]	2024	China	January 2024	Low back pain	41.60	50	1059	20
Hao et al [21]	2024	United States	April 2023	Neck pain	39.48	—^a^	243	6
Guo et al [22]	2024	Malaysia	September 2023	Knee joint pain	58.10	59	473	9
Ye et al [23]	2023	Canada	November 2022	Neck pain	41.14	59	192	5
Kantha et al [24]	2023	China	December 2021	Chronic musculoskeletal disorders	32-68	—	554	9
Guo et al [25]	2023	China	October 2022	Neck pain	41.12	54	382	8
Peng et al [26]	2022	China	May 2021	Arthroplasty	67.64	—	805	8
Huang et al [27]	2022	China	October 2020	Pain management	28.39	48	1947	31
Gazendam et al [28]	2022	Canada	October 2021	Arthroplasty	68.50	64	835	9
Bordeleau et al [29]	2022	United States	January 2019	Back pain	51.52	—	900	24
Cortés-Pérez et al [30]	2021	Spain	April 2021	Fibromyalgia syndrome in the female population	51.11	100	535	11
Wang et al [31]	2019	Australia	November 2018	Arthroplasty	65.68	59	2971	21
Gumaa and Rehan Youssef [32]	2019	Egypt	September 2018	Orthopedic rehabilitation	—	—	—	19

^a^Not applicable.

**Table 2:** Quality evaluation of included studies^a^.

Study	Q1	Q2	Q3	Q4	Q5	Q6	Q7	Q8	Q9	Q10	Q11	Q12	Q13	Q14	Q15	Q16	Overall rating
Su et al [19]	Yes	Yes	Yes	Yes	Yes	Yes	Yes	Yes	Yes	Yes	Yes	Yes	Yes	Yes	Yes	Yes	High
Li et al [20]	Yes	Yes	Yes	Yes	Yes	Yes	Yes	Yes	Yes	Yes	Yes	Yes	Yes	Yes	Yes	Yes	High
Hao et al [21]	Yes	Yes	Yes	Yes	No	Yes	Yes	Yes	Yes	No	Yes	Yes	Yes	Yes	No	Yes	Moderate
Guo et al [22]	Yes	Yes	No	Yes	Yes	Yes	Yes	Yes	Yes	No	Yes	Yes	Yes	No	Yes	Yes	Low
Ye et al [23]	Yes	Yes	Yes	Yes	No	Yes	Yes	Yes	Yes	Yes	Yes	No	Yes	Yes	No	Yes	Moderate
Kantha et al [24]	Yes	Yes	Yes	Yes	Yes	Yes	Yes	Yes	Yes	Yes	Yes	Yes	Yes	Yes	Yes	Yes	High
Guo et al [25]	Yes	Yes	Yes	Yes	Yes	Yes	Yes	Yes	Yes	Yes	No	Yes	Yes	Yes	No	Yes	Moderate
Peng et al [26]	Yes	Yes	Yes	Yes	Yes	Yes	Yes	Yes	Yes	Yes	Yes	Yes	Yes	Yes	Yes	Yes	High
Huang et al [27]	Yes	Yes	Yes	Yes	Yes	Yes	Yes	Yes	Yes	Yes	Yes	Yes	Yes	Yes	Yes	Yes	High
Gazendam et al [28]	Yes	Yes	Yes	Yes	Yes	Yes	Yes	Yes	Yes	Yes	Yes	Yes	Yes	Yes	Yes	Yes	High
Bordeleau et al [29]	Yes	Yes	Yes	Yes	Yes	Yes	Yes	Yes	Yes	No	Yes	Yes	No	Yes	Yes	Yes	Moderate
Cortés-Pérez et al [30]	Yes	No	Yes	Yes	No	Yes	Yes	Yes	Yes	Yes	No	Yes	Yes	Yes	Yes	Yes	Moderate
Wang et al [31]	Yes	Yes	No	Yes	Yes	Yes	Yes	Yes	Yes	Yes	Yes	Yes	No	Yes	Yes	Yes	Low
Gumaa and Rehan Youssef [32]	Yes	Yes	Yes	Yes	Yes	Yes	Yes	Yes	Yes	Yes	Yes	Yes	Yes	Yes	Yes	Yes	High

^a^The Measurement Tool to Assess Systematic Reviews (AMSTAR) criteria are Q1: Did the research questions and inclusion criteria for the review include the components of PICO (population or patient or problem, intervention, comparison, and outcome)? Q2: Did the report of the review contain an explicit statement that the review methods were established prior to the conduct of the review and did the report justify any significant deviations from the protocol? Q3: Did the review authors explain their selection of the study designs for inclusion in the review? Q4: Did the review authors use a comprehensive literature search strategy? Q5: Did the review authors perform study selection in duplicate? Q6: Did the review authors perform data extraction in duplicate? Q7: Did the review authors provide a list of excluded studies and justify the exclusions? Q8: Did the review authors describe the included studies in adequate detail? Q9: Did the review authors use a satisfactory technique for assessing the risk of bias (RoB) in individual studies that were included in the review? Q10: Did the review authors report on the sources of funding for the studies included in the review? Q11: If meta-analysis was performed, did the review authors use appropriate methods for statistical combination of results? Q12: If meta-analysis was performed, did the review authors assess the potential impact of RoB in individual studies on the results of the meta-analysis or other evidence synthesis? Q13: Did the review authors account for RoB in primary studies when interpreting or discussing the results of the review? Q14: Did the review authors provide a satisfactory explanation for, and discussion of, any heterogeneity observed in the results of the review? Q15: If they performed quantitative synthesis did the review authors carry out an adequate investigation of publication bias (small study bias) and discuss its likely impact on the results of the review? Q16: Did the review authors report any potential sources of conflict of interest, including any funding they received for conducting the review?

### Results of Umbrella Review

#### Knee Joint Pain

Only one study reported knee joint pain [22]. The type of VR that Guo et al [22] mainly aimed at is immersive. The findings of the study by Guo et al [22] show that immersive VR can reduce pain (MD –1.38, 95% CI –2.32 to –0.44; *P*=.004; *I*^2^=94%) in individuals with knee joint pain compared to traditional rehabilitation, and individuals who use immersive VR for rehabilitation have better balance (MD 0.41, 95% CI 0.12-0.69; *P*=.005; *I*^2^=0%). However, there were no statistically significant differences between immersive VR and traditional rehabilitation in terms of walking velocity (MD 0.04, 95% CI –0.22 to 0.29; *P*=.77; *I*^2^=21%) and ROM (MD 0.00, 95% CI –0.76 to 0.76; *P*>.99; *I*^2^=81%). Specific data can be found in Multimedia Appendix 4. Overall, VR has limited ability to improve the actual structure or function of tissues around the knee joint, which results in no significant difference in the improvement of ROM and walking velocity compared to traditional rehabilitation methods.

#### Fibromyalgia Syndrome in the Female Population

Only one study reported fibromyalgia syndrome in the female population [30]. Cortés-Pérez et al [30] included both immersive and nonimmersive VR studies. The findings of the study by Cortés-Pérez et al [30] show that VR is an effective treatment to reduce pain (SMD –0.45, 95% CI –0.70 to –0.20; *P*<.001), fatigue (SMD –0.58, 95% CI –1.01 to –0.14; *P*=.01), anxiety (SMD 0.50, 95% CI –0.908 to –0.029; *P*=.04), and depression (SMD 0.02, 95% CI –0.76 to –0.15; *P*=.003) associated with fibromyalgia syndrome. In addition, VR can improve dynamic balance (SMD –0.76, 95% CI –1.12 to –0.40; *P*<.001) and quality of life (SMD 0.13, 95% CI 0.30-0.81; *P*<.001) in patients with fibromyalgia syndrome. Specific data can be found in Multimedia Appendix 4.

#### Back Pain

**Figure 2:**
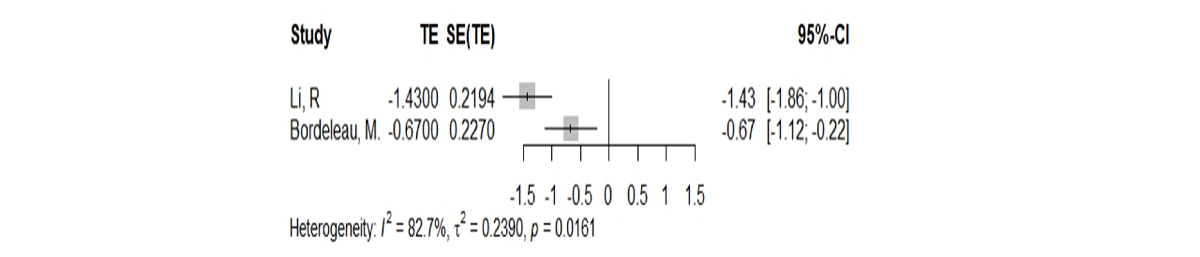
Forest plot of visual analog scale outcomes in patients with back pain [20,30]. TE: treatment effect.

Two meta-analyses reported on back pain [20,29]. Regarding how VR helps people with back discomfort, there was an overlap between the 2 meta-analyses [20,29]. GROOVE tool was used to identify the overlap of these 2 meta-analyses, and the specific results are shown in Multimedia Appendix 3. Based on the strategies for solving overlapping problems mentioned in the Overlapping Discovery and Processing section, the results of the Li et al [20] study are considered to represent the best available evidence. Li et al [20] included both immersive and nonimmersive VR studies. The results showed that VR can reduce the immediate pain (MD –1.43, 95% CI –1.86 to –1.00; *P*<.001; *I*^2^=95%) of patients with back pain (Figure 2). Specific data can be found in Multimedia Appendix 4.

#### Neck Pain

**Figure 3:**
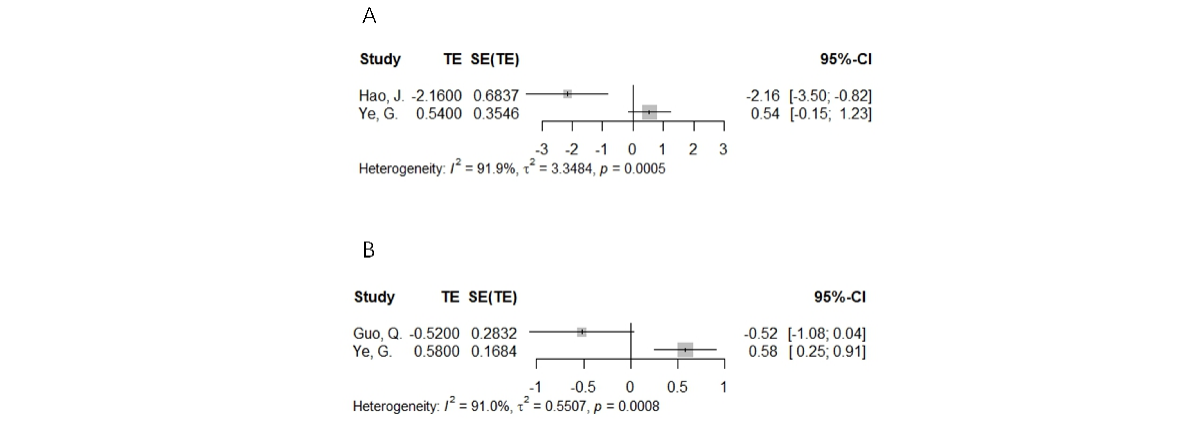
(A) Forest plot of Neck Disability Index outcomes in patients with neck pain. (B) Forest plot of visual analog scale outcomes in patients with neck pain [21,23,25]. TE: treatment effect.

Three meta-analyses reported on neck pain [21,23,25]. The research of Hao et al [21], Ye et al [23], and Guo et al [25] is mainly aimed at the research of immersive VR. The findings of the study by Hao et al [21] show that compared to traditional rehabilitation, immersive VR did not significantly reduce pain intensity (MD –0.61, 95% CI –1.27 to 0.05; *P*=.16; *I*^2^=41%) or improve fear of movement (MD –2.10, 95% CI –5.46 to –1.25; *P*=.13; *I*^2^=52%) in patients with neck pain. The findings of the study by Ye et al [23] show that the ROM (SMD 0.38, 95% CI –0.30 to 1.06; *P*=.27; *I*^2^=68%) in the immersive VR group was not significantly different from that of the group receiving conventional therapy. Regarding the effects of immersive VR on the VAS score of patients with neck pain, there was an overlap between 2 meta-analyses [23,25]. GROOVE tool was used to identify the overlap of these 2 meta-analyses, and the specific results are shown in Multimedia Appendix 3. Based on the strategies for solving overlapping problems mentioned in the Overlapping Discovery and Processing section, the results of the Guo et al [25] study are considered to represent the best available evidence. The results showed that immersive VR was not statistically different from traditional rehabilitation in reducing VAS scores (SMD –0.52, 95% CI –1.08 to 0.03; *P*=.002; *I*^2^=69%) in patients with neck pain (Figure 3). In addition, the Neck Disability Index results of Hao et al [21] and Ye et al [23] also overlap. After analysis, the study by Hao et al [21] is considered to be the best available evidence. The outcomes demonstrated that in terms of lowering the neck disability score, the VR group outperformed the traditional rehabilitation group (MD –2.16, 95% CI –3.50 to –0.82; *P*=.32; *I*^2^=15%). Specific data can be found in Multimedia Appendix 4.

#### Arthroplasty

**Figure 4:**
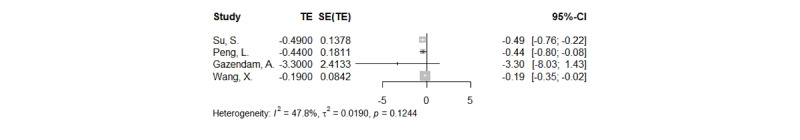
Forest plot of visual analog scale outcomes in patients who underwent arthroplasty [19,26,29,32]. TE: treatment effect.

Four meta-analyses reported on arthroplasty [19,26,28,31]. The type of VR that Peng et al [26] mainly aimed at is immersive. The other 3 studies included both immersive and nonimmersive VR studies. The findings of the study by Wang et al [31] show that VR was not statistically different from traditional rehabilitation in a 6-minute walk test (MD 29.36, 95% CI –6.99 to 65.71; *P*=.11; *I*^2^=88%) in patients who underwent arthroplasty. The findings of the study by Peng et al [26] show that the immersive VR group was better than the traditional rehabilitation group in Hospital for Special Surgery Knee Score (MD 8.30, 95% CI 6.92-9.67; *P*<.001; *I*^2^=24%). The results of the study by Su et al [19] show that there were no statistically significant variations in ROM between VR and traditional rehabilitation (MD 0.44, 95% CI –0.17 to 1.05; *P*=.16; *I*^2^=84%) in patients who underwent arthroplasty. The findings of the study by Su et al [19] show that the State-Trait Anxiety Inventory was lower in the VR group than the traditional rehabilitation group (MD –3.95, 95% CI –7.76 to –0.13; *P*=.04; *I*^2^=0%). Regarding the effects of VR on the VAS score of patients who underwent arthroplasty, there was an overlap between 4 meta-analyses [19,26,27,31] (Figure 4). The GROOVE tool was used to identify the overlap among these 4 meta-analyses, with specific results provided in Multimedia Appendix 3. Based on the strategies for addressing overlapping issues outlined in the Overlapping Discovery and Processing section, the study by Su et al [19] was deemed to provide the best available evidence. The results demonstrated that VR outperformed traditional rehabilitation in reducing VAS scores (SMD –0.49, 95% CI –0.76 to –0.22; *P*<.001; *I*^2^=46%). Furthermore, the overlap was identified in the WOMAC and TUG scores between the studies by Su et al [19] and Peng et al [26]. Following analysis, the study by Su et al [19] was determined to provide the most robust evidence. Their outcomes demonstrated that the VR group showed greater improvement in WOMAC scores compared to the traditional rehabilitation group (MD –0.65, 95% CI –0.92 to –0.38; *P*<.001; *I*^2^=0%), while the TUG scores did not differ significantly (MD –1.13, 95% CI –3.52 to 0.86; *P*=.24; *I*^2^=0%). Detailed data can be found in Multimedia Appendix 4.

#### Other Musculoskeletal Disorders

The study by Gumaa and Rehan Youssef [32] showed that, compared to traditional rehabilitation, VR was not statistically different from traditional rehabilitation in reducing VAS scores (SMD –0.24, 95% CI –0.61 to 0.12; *P*=.19; *I*^2^=0%) in patients with orthopedic rehabilitation. The study’s findings by Huang et al [27] show that the immersive VR group was better than the traditional rehabilitation group in reducing pain intensity (weighted mean difference –1.62, 95% CI –1.86 to –1.38; *P*=.095; *I*^2^=27%). The study’s findings by Kantha et al [24] show that the VR group was better than the traditional rehabilitation group in lowering discomfort in those with long-term musculoskeletal conditions (MD –8.09, 95% CI –12.42 to –3.76; *P*<.001; *I*^2^=0%). However, there was no statistically significant difference in psychological distress (MD –0.07, 95% CI –0.45 to 0.32; *P*=.59; *I*^2^=0%) and functional disability (MD 0.14, 95% CI –0.12 to 0.39; *P*=.29; *I*^2^=0%).

## Discussion

### Principal Findings

This umbrella review highlights the potential of VR in the rehabilitation of various musculoskeletal disorders. In cases of knee joint pain, VR significantly reduces pain and improves balance but shows no significant difference compared to traditional rehabilitation in terms of walking speed and ROM. For female patients with fibromyalgia syndrome, VR treatment effectively reduces pain, fatigue, anxiety, and depression while improving dynamic balance and quality of life. In patients with back pain, VR can reduce immediate pain-related fears and improve disability outcomes. As for neck pain treatment, VR does not significantly lessen pain intensity or enhance ROM. For individuals undergoing arthroplasty, VR shows no significant impact on the 6-minute walk test but outperforms traditional rehabilitation in improving the Hospital for Special Surgery score and reducing state-trait anxiety levels. Finally, 7 meta-analyses investigated the efficacy of immersive VR, revealing its potential in reducing pain and improving specific functional outcomes, particularly for conditions such as knee pain, fibromyalgia, and back pain. To translate these results into practical applications, clinicians should consider integrating VR into treatment plans tailored for specific conditions like knee joint pain. This can be achieved by using VR alongside traditional therapies to provide a comprehensive rehabilitation approach. Additionally, clinicians should leverage VR systems with real-time monitoring capabilities to adjust treatment plans based on patient performance. For patients, VR can be seamlessly incorporated into daily routines for pain management through distraction or relaxation techniques as well as for maintaining mobility.

The primary contribution of this study is its aggregation of high-quality evidence from multiple sources, offering a comprehensive understanding of the efficacy of treatments such as VR rehabilitation in managing conditions like knee joint pain, fibromyalgia, and chronic neck pain. A key strength of this work lies in its rigorous methodology, which involved a meticulous selection process of meta-analyses to ensure that only high-quality evidence was included. Additionally, this study evaluated outcomes using the GRADE system, providing an objective assessment of the strength of evidence across various interventions. By incorporating a diverse range of musculoskeletal disorders and treatment modalities, this umbrella review offers clinicians a holistic view of the effectiveness of current interventions, which is essential for guiding clinical decision-making.

### Comparison to Prior Work

This study’s findings were compared with those of previous studies. Viderman et al [34] demonstrated VR’s analgesic efficacy in perioperative and chronic pain settings, reinforcing the hypothesis that VR operates as a potent distractor by modulating pain signaling pathways and reducing nociceptive stimuli. Consistent with this, Goudman et al [35] emphasized that VR interventions significantly reduce pain and improve psychological outcomes, including depression and anxiety, in chronic pain settings. These outcomes resonate with our observations of VR’s role in not only physical recovery but also emotional well-being, particularly in populations with fibromyalgia and chronic low back pain. Bilika et al [36] highlighted the efficacy of VR-based exercise therapy in addressing chronic musculoskeletal pain through enhanced patient engagement and increased treatment satisfaction. The incorporation of immersive environments creates a motivating and enjoyable rehabilitation experience, which could explain the consistent adherence and improvement rates observed in various studies. This suggests a dual advantage: functional recovery and psychological upliftment. Findings by Austin [37] highlight the analgesic effects of VR in perioperative and chronic pain settings, suggesting that VR works by modulating pain signaling pathways and reducing notional sensory stimuli. This is consistent with the findings in this review. The results of the study by Lo et al [38] highlight that VR interventions significantly reduce pain and improve psychological outcomes, including depression and anxiety, in chronic pain settings. This is consistent with the findings in this review regarding fibromyalgia syndrome, where VR treatment effectively reduces pain, fatigue, anxiety, and depression while improving homeostasis and quality of life. Research by Guerra-Armas et al [39] reported the efficacy of VR-based exercise therapy in addressing chronic musculoskeletal pain. This is highly consistent with the results reported in this study.

The observed difference in the efficacy of VR for neck pain compared to conditions such as knee pain or fibromyalgia can be explained by several different mechanisms: neck pain often involves complex biomechanical interactions among the cervical vertebrae, muscles, and ligaments. VR interventions might not adequately replicate the necessary ROM or force vectors required for the effective rehabilitation of cervical structures [40]. In contrast, knee joint pain, which involves a more straightforward joint mechanism, might benefit more readily from VR exercises simulating weight-bearing, balance, and proprioceptive training [41]. Neck pain can be multifactorial, often involving neuropathic components, muscle tension, or referred pain from other regions. The pain mechanisms in fibromyalgia, characterized by widespread pain and hyperalgesia, or knee joint pain, often associated with mechanical issues, differ significantly. VR interventions designed to address pain through distraction, relaxation, or cognitive behavioral approaches might not effectively target the specific pain pathways involved in neck pain [42]. The effectiveness of VR in rehabilitation is highly dependent on the precision and specificity of the virtual environment. For neck pain, VR programs need to incorporate specialized simulations focusing on cervical mobility, postural correction, and perhaps even real-time biofeedback for muscle tension [43]. While VR-based interventions demonstrate substantial benefits for certain musculoskeletal disorders, such as knee pain and fibromyalgia, these advantages may not always extend to all aspects of rehabilitation. In contrast, more conventional interventions, such as physical therapy or pharmacologic treatments, may still be necessary, particularly when addressing pain or severe functional limitations that VR may not effectively mitigate. Furthermore, although the reviewed studies indicate that VR can be a valuable tool in managing musculoskeletal disorders, it is crucial to recognize the limitations of these interventions. These limitations include the need for specialized equipment, potential patient discomfort with technology, and the higher costs associated with VR systems compared to traditional rehabilitation methods.

To effectively integrate VR into rehabilitation, clinicians should tailor interventions to specific conditions, such as using VR for balance exercises in patients with knee pain or relaxation techniques for fibromyalgia. Combining VR with other therapies, like physical therapy, can provide real-time feedback, thereby enhancing patient adherence and treatment effectiveness. Regular monitoring through VR tracking systems and follow-up assessments will ensure that progress is tracked in terms of mobility, pain, and quality of life. Additionally, clinicians should educate patients on VR use through training sessions and offer ongoing support. For patients, incorporating VR into daily life involves setting aside time for short, daily sessions focused on rehabilitation exercises or pain management through virtual relaxation environments. Engaging with gamified VR programs can maintain motivation by tracking progress and offering rewards. Patients need a dedicated, obstacle-free space for VR sessions and should take breaks to prevent physical discomfort, ensuring a beneficial and enjoyable VR experience that complements their rehabilitation journey.

### Strengths and Limitations

This umbrella review had several limitations. First, individual meta-analyses inherently carry biases, which may affect the overall findings. Second, many primary studies included in the review lacked detailed outcome assessments or follow-up information, limiting the reliability of their conclusions. Third, the evidence currently available must be updated, as several high-quality RCTs have been published recently. Fourth, this review only included meta-analyses published in English, potentially excluding relevant studies in other languages. Fifth, the study did not categorize the different types of VR interventions, which could have provided a more nuanced understanding of their effectiveness. Finally, the findings of this review require validation through large-scale, rigorously designed clinical trials to confirm their broad applicability and assess their long-term effects.

### Future Directions

Further studies are needed to identify patient characteristics that predict successful outcomes with VR and other interventions. For example, age, severity of the condition, and comorbidities may influence how well a patient responds to VR rehabilitation [44]. Personalized treatment protocols based on these factors could enhance the effectiveness of interventions. Given the higher costs associated with VR systems, future research should explore the cost-effectiveness of VR rehabilitation compared to traditional methods and conduct large-scale RCTs for different types of VR interventions [39].

### Conclusions

VR demonstrates significant potential in the rehabilitation of various musculoskeletal conditions. It effectively alleviates pain, enhances psychological well-being, and facilitates the recovery of motor function in patients.

## Appendix

Multimedia Appendix 1 PRIOR checklist.

Multimedia Appendix 2 Search strategy.

Multimedia Appendix 3 Results of Graphical Representation of Overlap for Overviews overlap analysis.

Multimedia Appendix 4 Association between the virtual reality and musculoskeletal disease outcomes.

Multimedia Appendix 5 Excluded studies and their associated reasons.

Multimedia Appendix 6 Main conclusions of the study.

## Abbreviations

AMSTAR 2: Measurement Tool to Assess Systematic Reviews 2
GRADE: Grading of Recommendations Assessment, Development, and Evaluation
GROOVE: Graphical Representation of Overlap for Overviews
MD: mean difference
PRISMA: Preferred Reporting Items for Systematic Reviews and Meta-Analyses
RCT: randomized controlled trial
ROM: range of motion
SMD: standardized mean difference
TUG: timed up and go test
VAS: visual analog scale
VR: virtual reality
WOMAC: Western Ontario and McMaster Universities Osteoarthritis Index
