# Supplementary material for: The Efficacy of Virtual Reality on the Rehabilitation of Musculoskeletal Diseases: Umbrella Review
Source: J Med Internet Res. 2025 Apr 25;27:e64576. doi: 10.2196/64576 (PMC12064964; doi:10.2196/64576)
Supplement: Multimedia Appendix 2 [file jmir_v27i1e64576_app2.docx]

## Pubmed：

(((((Meta-Analysis[MeSH Terms]) OR (Meta-Analysis as Topic[MeSH Terms])) OR (((((((((Meta-Analysis[Title/Abstract]) OR (Meta-Analysis as Topic[Title/Abstract])) OR (Meta Analysis as Topic[Title/Abstract])) OR (Data Pooling[Title/Abstract])) OR (Data Poolings[Title/Abstract])) OR (Overviews, Clinical Trial[Title/Abstract])) OR (Clinical Trial Overviews[Title/Abstract])) OR (Clinical Trial Overview[Title/Abstract])) OR (Overview, Clinical Trial[Title/Abstract]))) OR (Systematic Reviews as Topic[MeSH Terms])) OR (((((Systematic Review[Title/Abstract]) OR (Systematic Reviews as Topic[Title/Abstract])) OR (Systematic Review as Topic[Title/Abstract])) OR (Reviews Systematic as Topic[Title/Abstract])) OR (Umbrella Reviews as Topic[Title/Abstract]))) AND (((Virtual Reality[MeSH Terms]) OR (((((((((((((Virtual Reality[Title/Abstract]) OR (Reality, Virtual[Title/Abstract])) OR (Virtual Reality, Educational[Title/Abstract])) OR (Educational Virtual Realities[Title/Abstract])) OR (Educational Virtual Reality[Title/Abstract])) OR (Reality, Educational Virtual[Title/Abstract])) OR (Virtual Realities, Educational[Title/Abstract])) OR (Virtual Reality, Instructional[Title/Abstract])) OR (Instructional Virtual Realities[Title/Abstract])) OR (Instructional Virtual Reality[Title/Abstract])) OR (Realities, Instructional Virtual[Title/Abstract])) OR (Reality, Instructional Virtual[Title/Abstract])) OR (Virtual Realities, Instructional[Title/Abstract]))) AND (((("Musculoskeletal Diseases"[Mesh]) OR ("Orthopedics"[Mesh])) OR (Orthopedics[Title/Abstract])) OR ((((Musculoskeletal Diseases[Title/Abstract]) OR (Musculoskeletal Disease[Title/Abstract])) OR (Orthopedic Disorders[Title/Abstract])) OR (Orthopedic Disorder[Title/Abstract]))))

## Embase

#12. #9 AND #10 AND #11

#11. #1 OR #2

#10. #3 OR #4 OR #5

#9. #6 OR #7 OR #8

#8. 'meta analysis':ab,ti OR 'systematic

review':ab,ti

#7. 'systematic review'/exp OR 'systematic review'

#6. 'meta analysis'/exp OR 'meta analysis'

#5. 'musculoskeletal diseases':ab,ti OR

'musculoskeletal disease':ab,ti OR 'orthopedic

disorders':ab,ti OR 'orthopedic disorder':ab,ti

OR orthopedics:ab,ti

#4. 'orthopedics'/exp OR 'orthopedics'

#3. 'musculoskeletal disease'/exp OR 'musculoskeletal

disease'

#2. 'virtual reality':ab,ti OR 'reality,

virtual':ab,ti OR 'virtual reality,

educational':ab,ti OR 'educational virtual

realities':ab,ti OR 'educational virtual

reality':ab,ti OR 'reality, educational

virtual':ab,ti OR 'virtual realities,

educational':ab,ti OR 'virtual reality,

instructional':ab,ti OR 'instructional virtual

realities':ab,ti OR 'instructional virtual

reality':ab,ti OR 'realities, instructional

virtual':ab,ti OR 'reality, instructional

virtual':ab,ti OR 'virtual realities,

instructional':ab,ti

#1. 'virtual reality'/exp OR 'virtual reality'

.......................................................

## Cochrane

#1 MeSH descriptor: [Meta-Analysis as Topic] explode all trees

#2 (Meta-Analysis as Topic):ti,ab,kw OR (Meta Analysis as Topic):ti,ab,kw OR (Data Pooling):ti,ab,kw OR (Data Poolings):ti,ab,kw OR (Overviews, Clinical Trial):ti,ab,kw 10970

#3 (Clinical Trial Overviews):ti,ab,kw OR (Clinical Trial Overview):ti,ab,kw OR (Overview, Clinical Trial):ti,ab,kw OR (Meta-Analysis):ti,ab,kw

#4 MeSH descriptor: [Systematic Reviews as Topic] explode all trees

#5 (Systematic Review):ti,ab,kw OR (Systematic Reviews as Topic):ti,ab,kw OR (Systematic Review as Topic):ti,ab,kw OR (Reviews Systematic as Topic):ti,ab,kw OR (Umbrella Reviews as Topic):ti,ab,kw

#6 MeSH descriptor: [Virtual Reality] explode all trees

#7 (Virtual Reality):ti,ab,kw OR (Reality, Virtual):ti,ab,kw OR (Virtual Reality, Educational):ti,ab,kw OR (Educational Virtual Realities):ti,ab,kw OR (Educational Virtual Reality):ti,ab,kw

#8 (Reality, Educational Virtual):ti,ab,kw OR (Virtual Realities, Educational):ti,ab,kw OR (Virtual Reality, Instructional):ti,ab,kw OR (Instructional Virtual Realities):ti,ab,kw OR (Instructional Virtual Reality):ti,ab,kw

#9 (Realities, Instructional Virtual):ti,ab,kw OR (Reality, Instructional Virtual):ti,ab,kw OR (Virtual Realities, Instructional):ti,ab,kw

#10 MeSH descriptor: [Musculoskeletal Diseases] explode all trees

#11 (Musculoskeletal Diseases):ti,ab,kw OR (Musculoskeletal Disease):ti,ab,kw OR (Orthopedic Disorders):ti,ab,kw OR (Orthopedic Disorder):ti,ab,kw

#12 MeSH descriptor: [Orthopedics] explode all trees

#13 (Orthopedics):ti,ab,kw

#14 #1 OR #2 OR #3 OR #4 OR #5

#15 #6 OR #7 OR #8 OR #9

#16 #10 OR #11 OR #12 OR #13

#17 #14 AND #15 AND #16 12
