# Supplementary material for: The Efficacy of Virtual Reality on the Rehabilitation of Musculoskeletal Diseases: Umbrella Review
Source: J Med Internet Res. 2025 Apr 25;27:e64576. doi: 10.2196/64576 (PMC12064964; doi:10.2196/64576)

1. Back pain


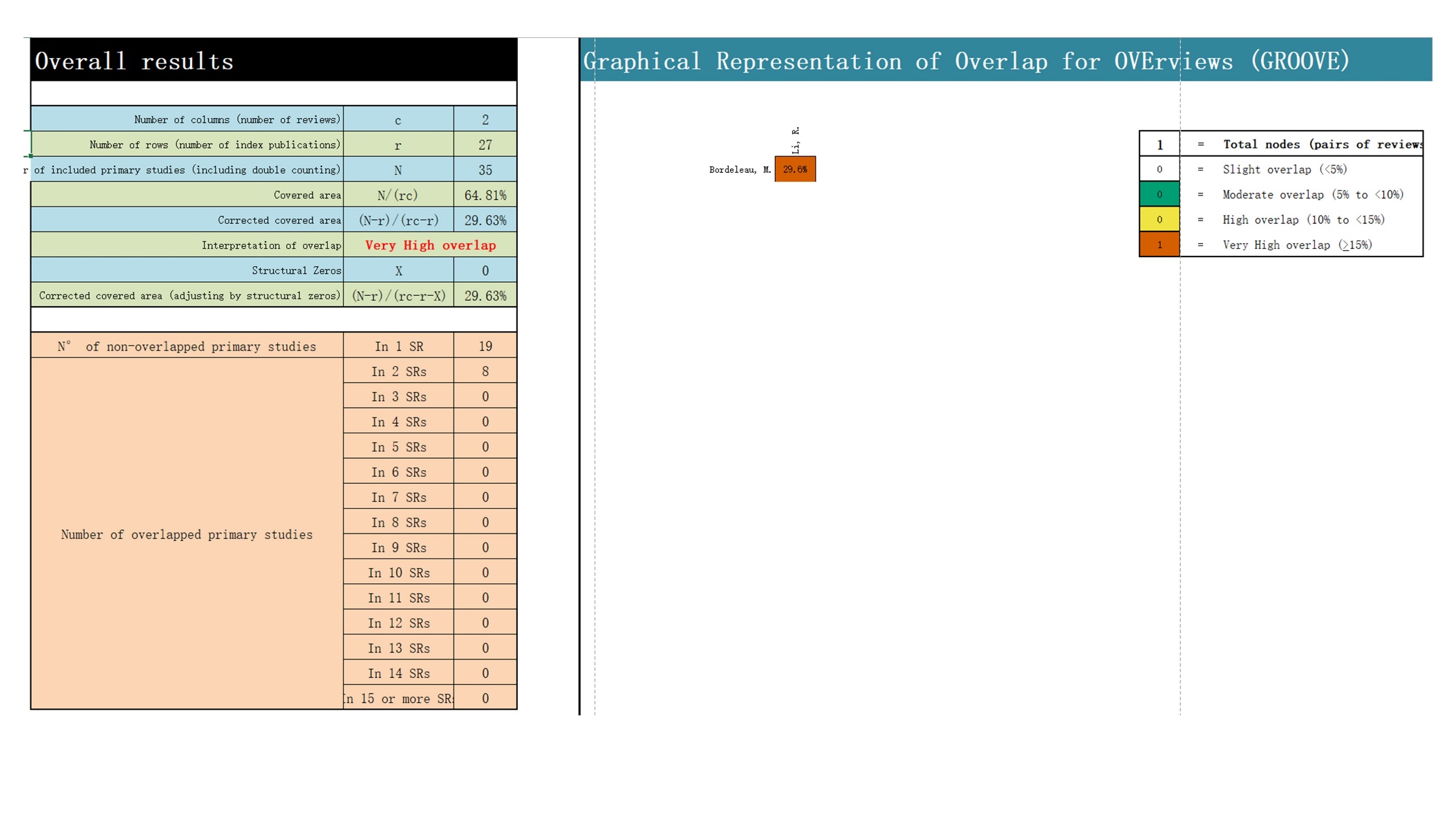


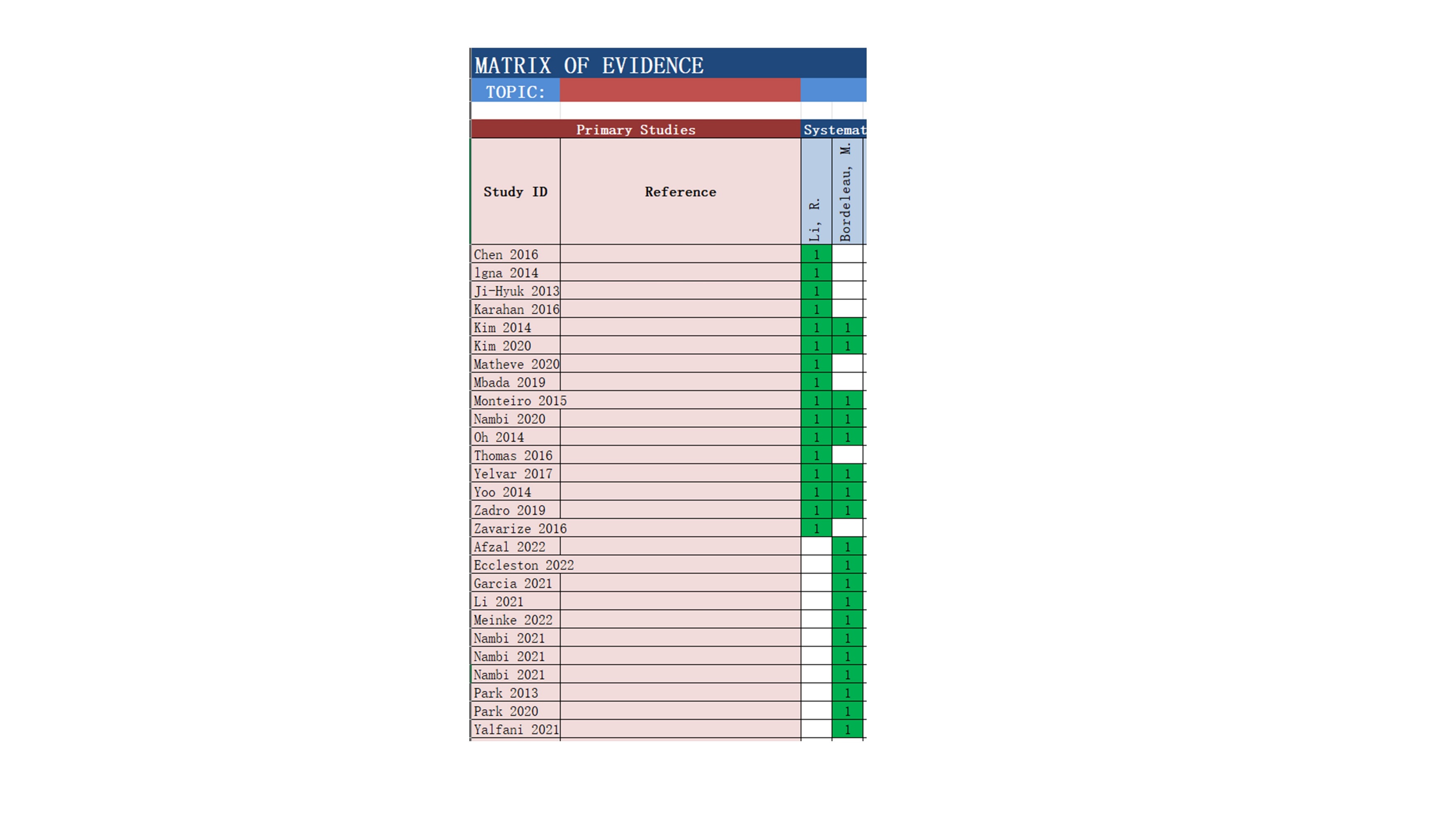


1. 1. Neck pain-VAS


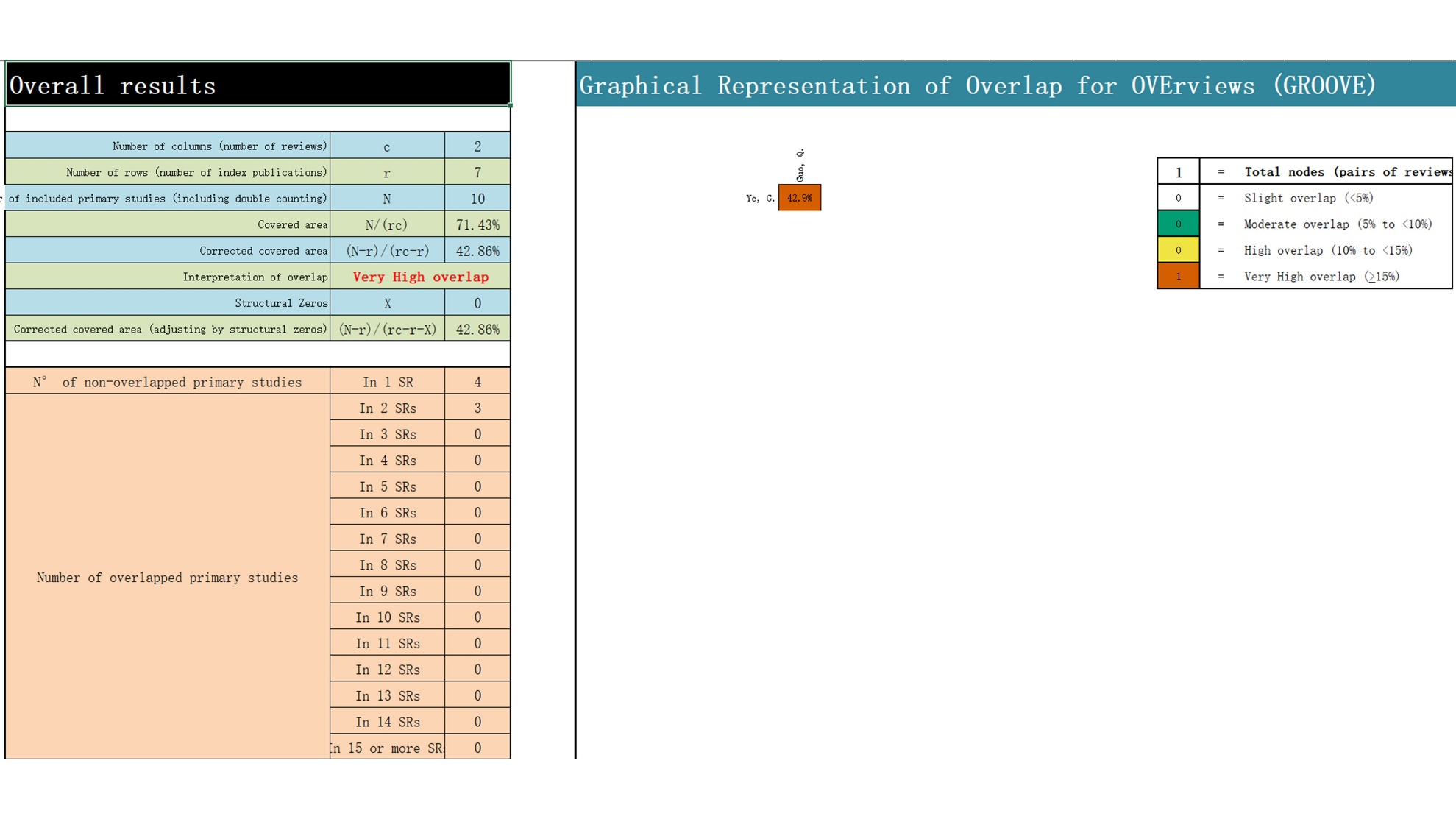


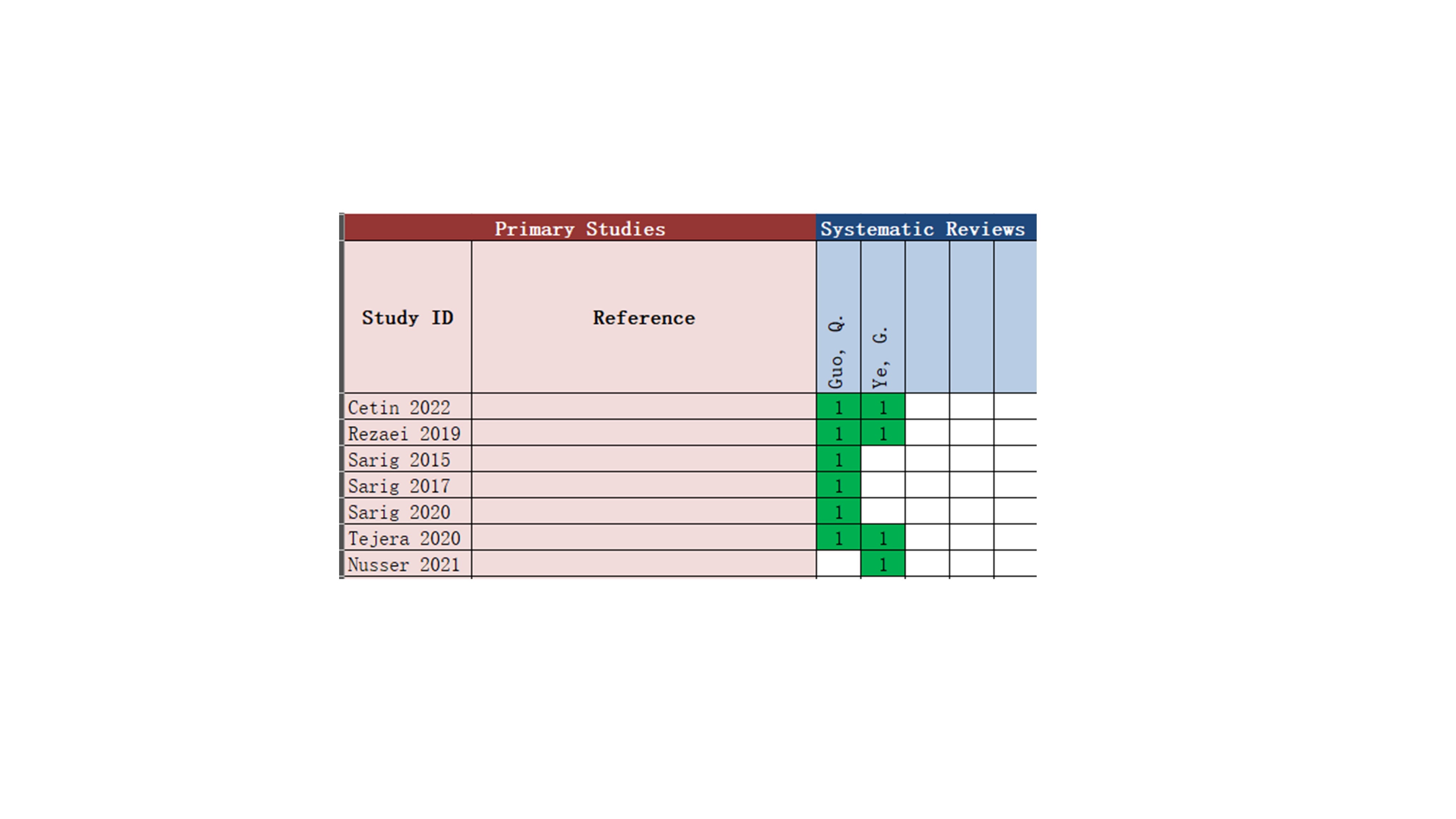


B.2. Neck pain-NDI


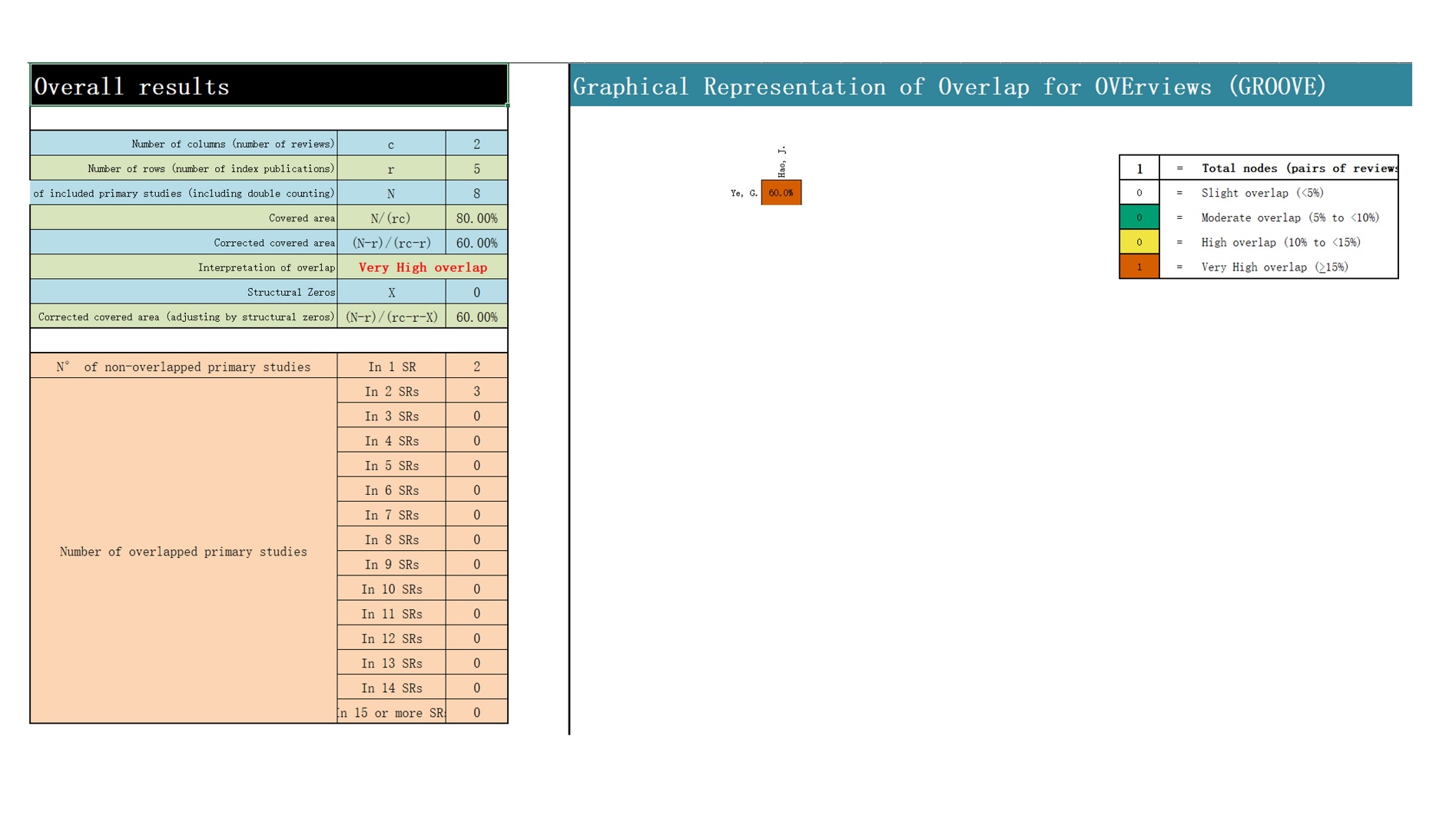


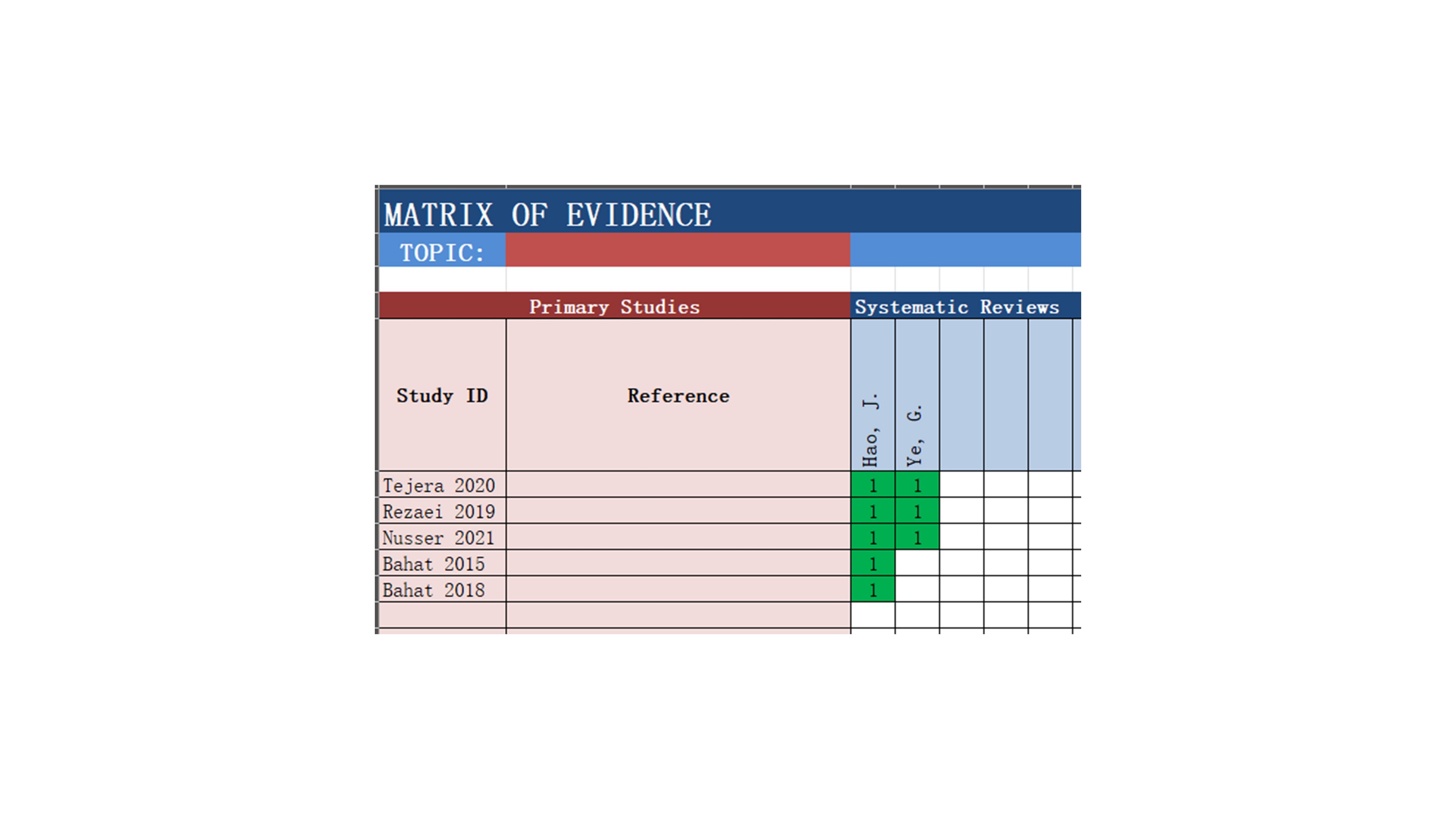


C.1. Arthroplasty-WOMAC


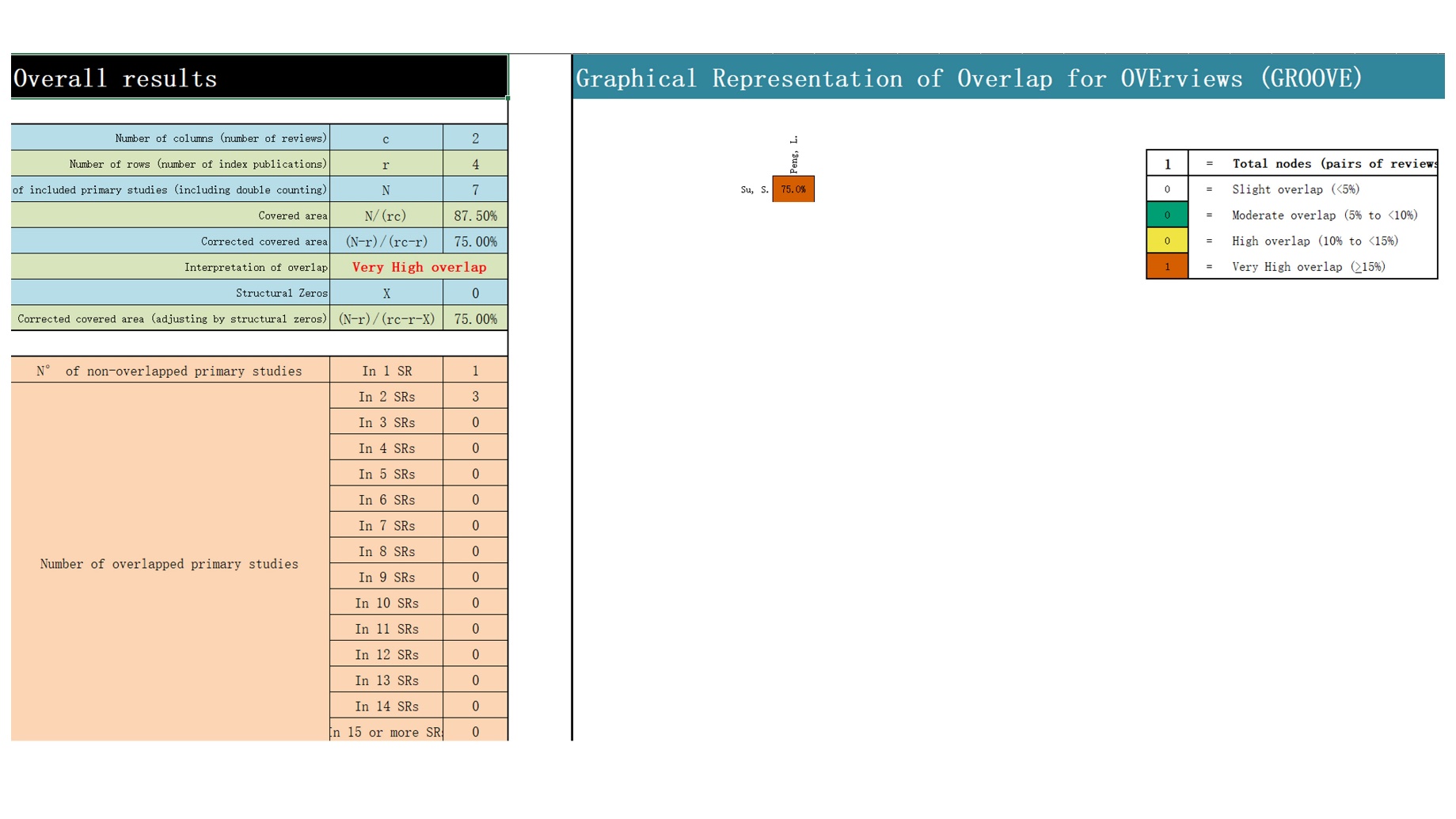


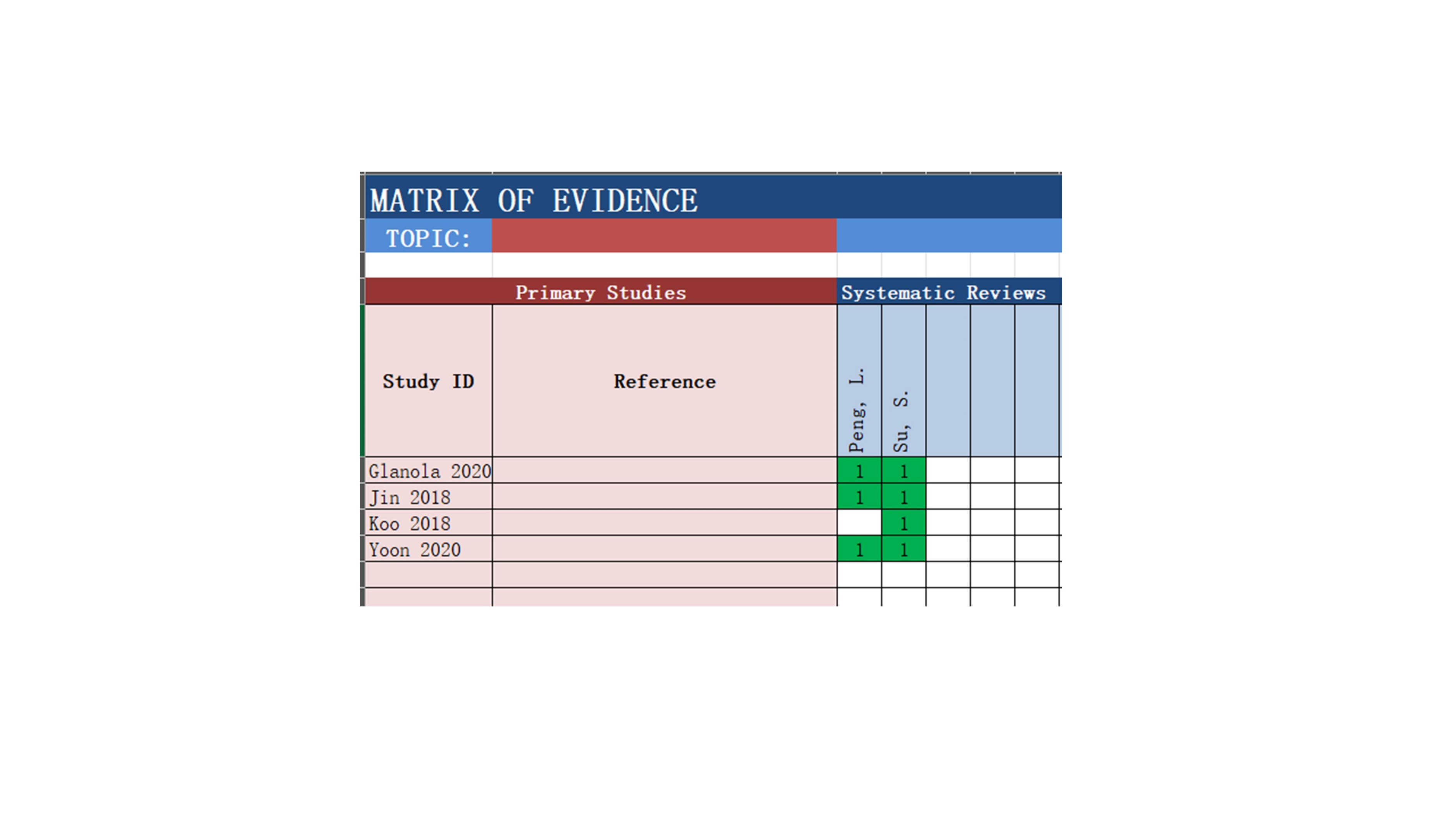


C.2. Arthroplasty-TUG


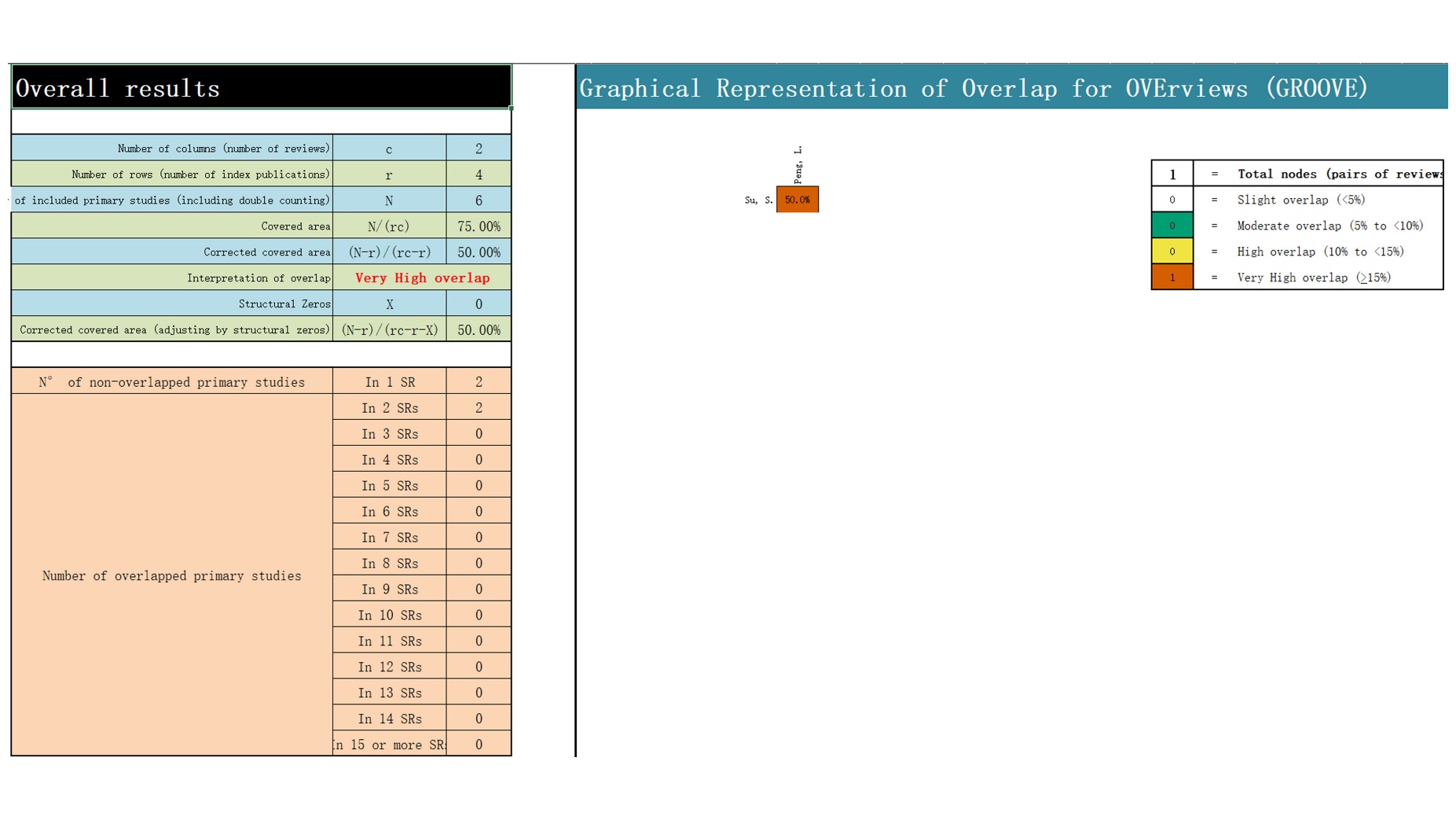


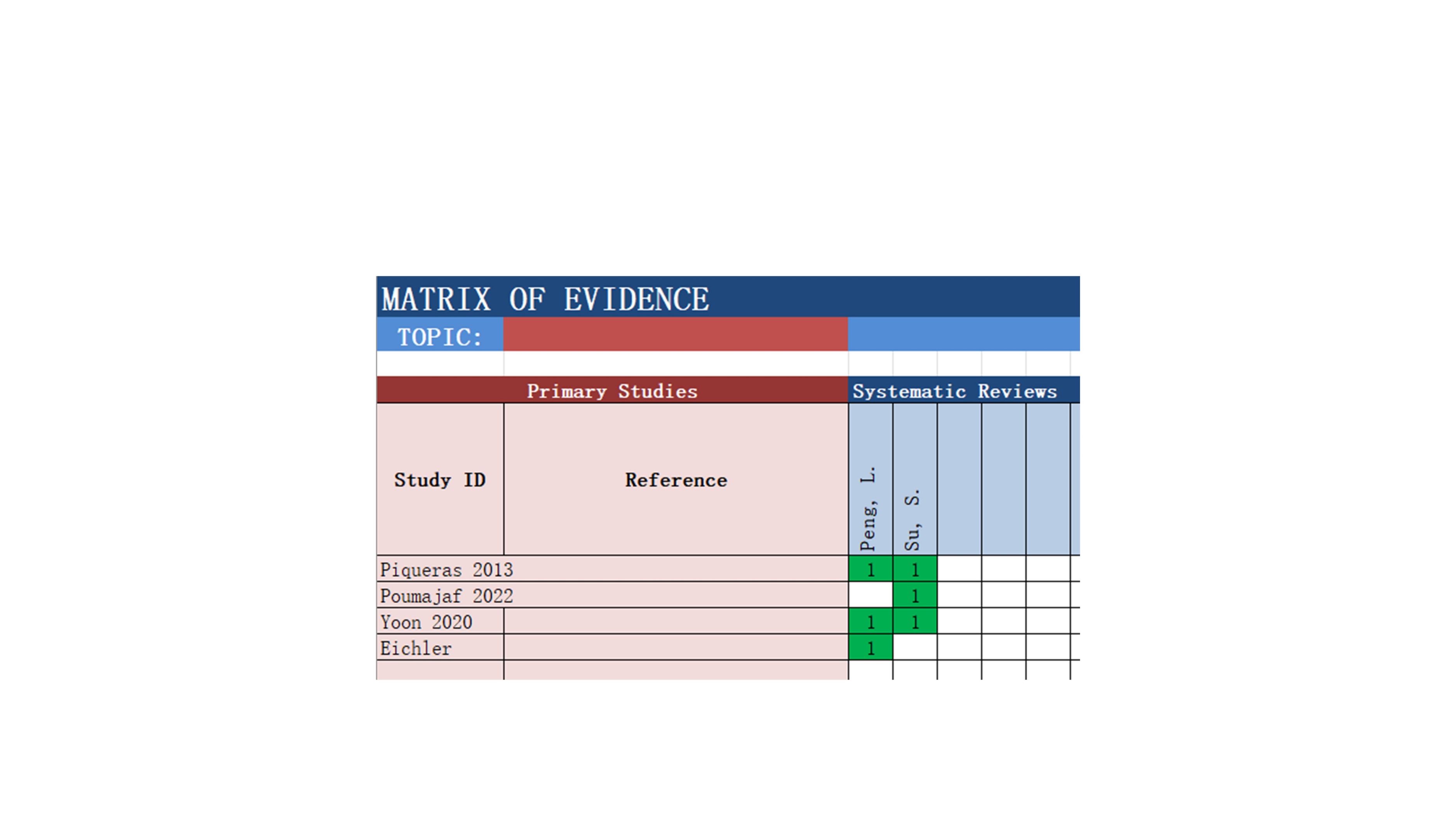


C.3. Arthroplasty-VAS


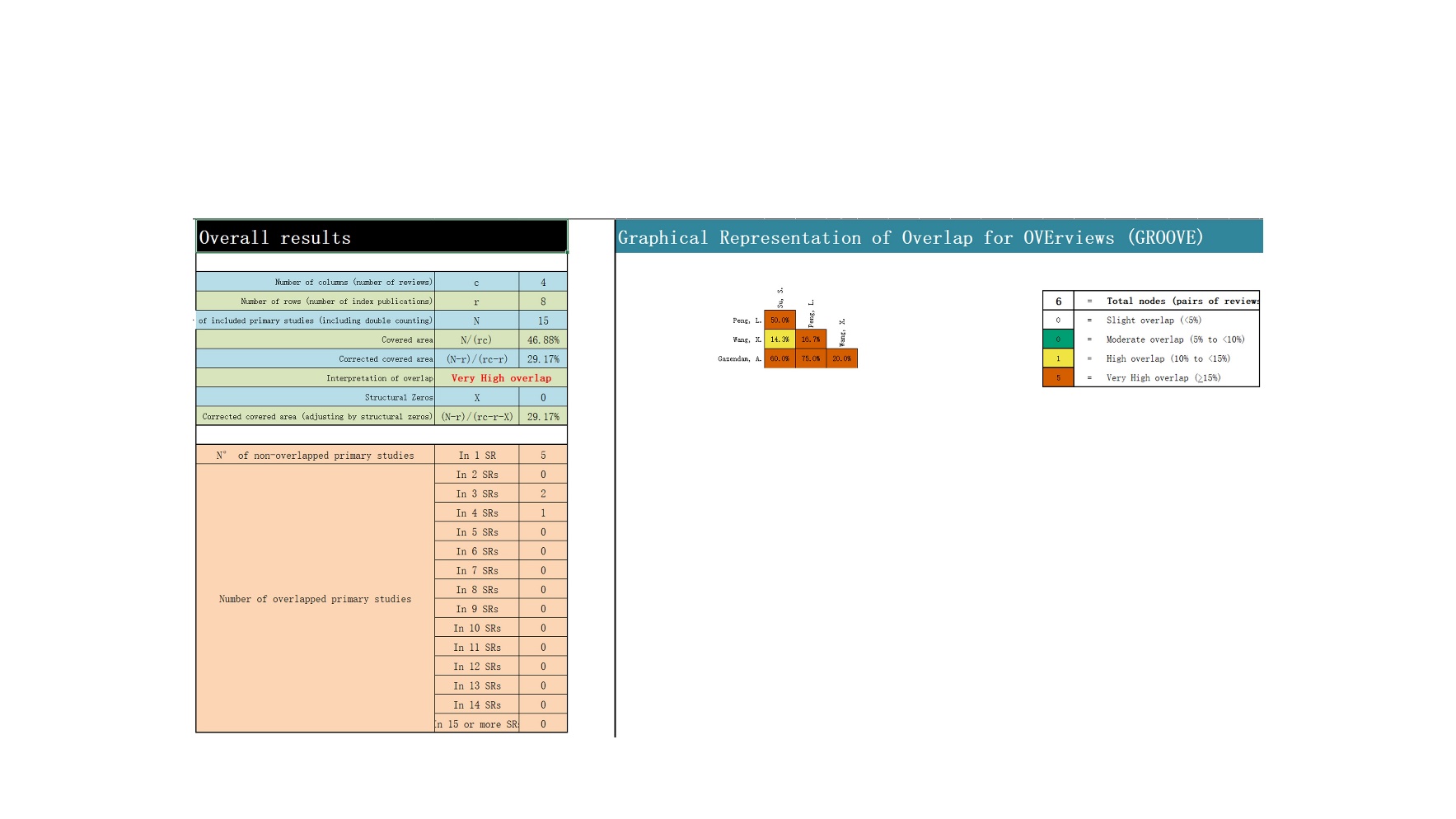


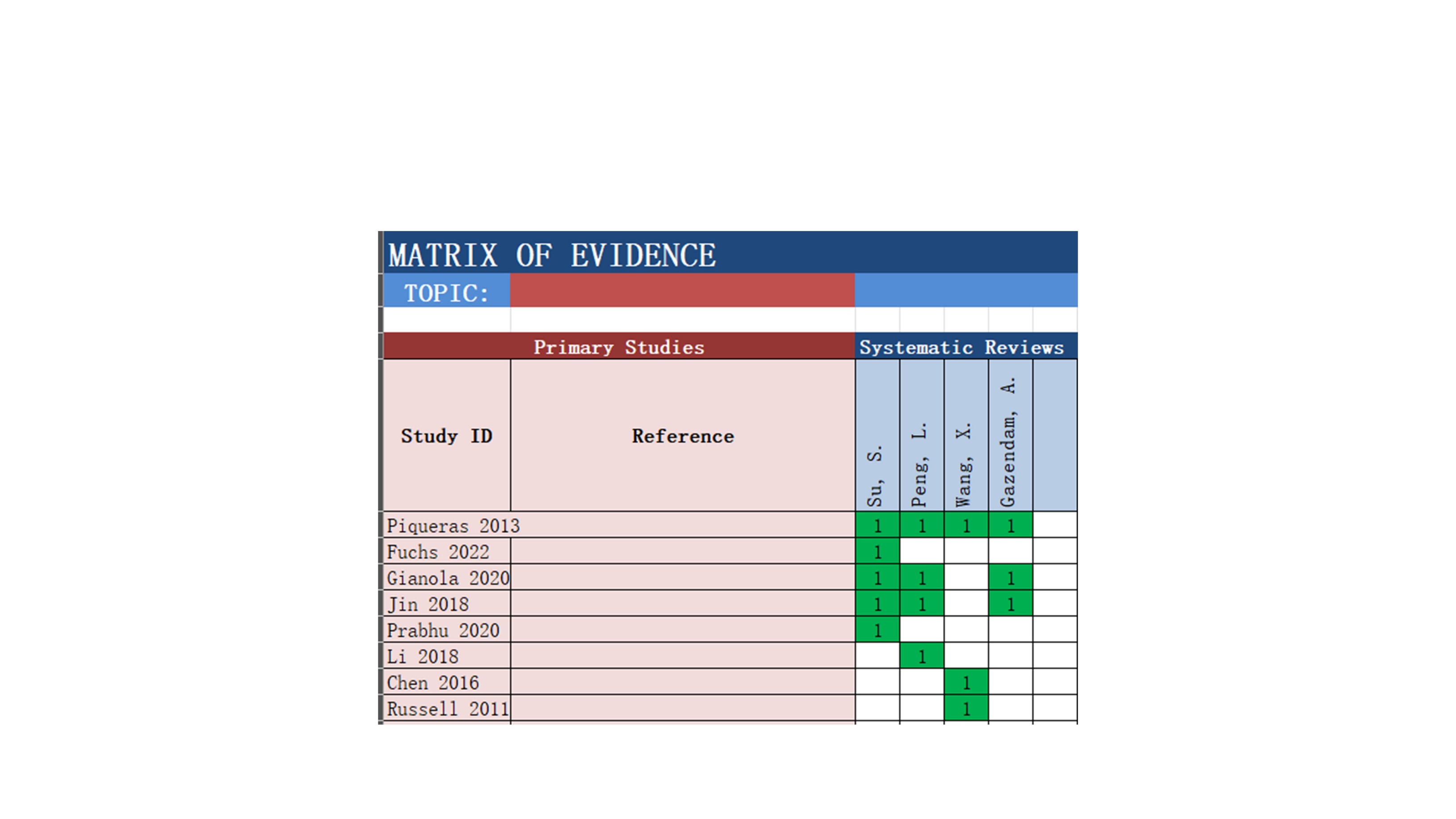

Supplement: Multimedia Appendix 3 [file jmir_v27i1e64576_app3.docx]
