# Supplementary material for: The Efficacy of Virtual Reality on the Rehabilitation of Musculoskeletal Diseases: Umbrella Review
Source: J Med Internet Res. 2025 Apr 25;27:e64576. doi: 10.2196/64576 (PMC12064964; doi:10.2196/64576)
Supplement: Multimedia Appendix 4 [file jmir_v27i1e64576_app4.docx]

| Author | Year | Condition | Outcome | No. of studies included | Type of metric | Effect | 95% CI | Effects model | I² | GRADE | Evidence class |
| --- | --- | --- | --- | --- | --- | --- | --- | --- | --- | --- | --- |
| Su et al [19] | 2024 | Arthroplasty | VAS | 5 | MD | -0.49 | -0.76 to -0.22 | Fixed | 48 | Low | IV |
| Su et al [19] | 2024 | Arthroplasty | WOMAC | 4 | MD | -0.65 | -0.92 to -0.38 | Random | 0 | Low | IV |
| Su et al [19] | 2024 | Arthroplasty | ROM | 4 | MD | 0.44 | -0.17 to 1.05 | Random | 84 | Low | NS |
| Su et al [19] | 2024 | Arthroplasty | TUG | 3 | MD | -1.33 | -3.52 to 0.86 | Fixed | 0 | Low | NS |
| Su et al [19] | 2024 | Arthroplasty | STAI | 2 | MD | -3.95 | -7.76 to -0.13 | Fixed | 0 | Very Low | IV |
| Li et al [20] | 2024 | Low Back Pain | VAS | 19 | MD | -1.43 | -1.86 to -1.00 | Random | 95 | Moderate | IV |
| Li et al [20] | 2024 | Low Back Pain | Pain-related fear | 6 | MD | -5.46 | -9.40 to -1.52 | Random | 90 | Low | IV |
| Li et al [20] | 2024 | Low Back Pain | Disability | 8 | MD | -11.50 | -20.00 to -3.01 | Random | 95 | Low | IV |
| Hao et al [21] | 2024 | Neck Pain | NDI | 5 | MD | -2.16 | -3.50 to -0.82 | Fixed | 15 | Low | IV |
| Hao et al [21] | 2024 | Neck Pain | Pain intensity | 4 | MD | -0.61 | -1.27 to 0.05 | Fixed | 41 | Low | NS |
| Hao et al [21] | 2024 | Neck Pain | Kinesiophobia | 3 | MD | -2.10 | -5.46 to 1.25 | Random | 51 | Low | NS |
| Guo et al [22] | 2024 | Knee joint pain | Balance | 4 | MD | 0.41 | 0.12 to 0.69 | Fixed | 0 | Low | IV |
| Guo et al [22] | 2024 | Knee joint pain | VAS | 7 | MD | -1.38 | -2.32 to -0.44 | Random | 94 | Low | IV |
| Guo et al [22] | 2024 | Knee joint pain | ROM | 3 | MD | 0.00 | -0.76 to 0.76 | Random | 81 | Low | NS |
| Guo et al [22] | 2024 | Knee joint pain | WV | 4 | MD | 0.04 | -0.22 to 0.29 | Fixed | 21 | Low | NS |
| Ye et al [23] | 2023 | Neck Pain | VAS | 4 | SMD | 0.58 | 0.25 to 0.91 | Random | 2 | Low | IV |
| Ye et al [23] | 2023 | Neck Pain | NDI | 3 | SMD | 0.54 | -0.15 to 1.24 | Random | 72 | Low | NS |
| Ye et al [23] | 2023 | Neck Pain | ROM | 3 | SMD | 0.38 | -0.30 to 1.06 | Random | 68 | Low | NS |
| Kantha et al [24] | 2023 | Chronic Musculoskeletal Disorders | VAS | 5 | MD | -8.09 | -12.42 to -3.76 | Fixed | 0 | Low | IV |
| Kantha et al [24] | 2023 | Chronic Musculoskeletal Disorders | Psychological distress | 2 | SMD | -0.07 | -0.45 to 0.32 | Fixed | 0 | Very Low | NS |
| Kantha et al [24] | 2023 | Chronic Musculoskeletal Disorders | Functional disability | 5 | SMD | 0.14 | -0.12 to 0.39 | Fixed | 0 | Low | NS |
| Guo et al [25] | 2023 | Neck Pain | VAS | 6 | SMD | -0.52 | -1.08 to 0.03 | Random | 78 | Low | NS |
| Peng et al [26] | 2022 | Arthroplasty | VAS | 7 | SMD | -0.44 | -0.79 to -0.08 | Random | 63 | Low | IV |
| Peng et al [26] | 2022 | Arthroplasty | WOMAC | 6 | SMD | -0.71 | -1.03 to -0.40 | Random | 0 | Low | IV |
| Peng et al [26] | 2022 | Arthroplasty | HHS | 5 | MD | 8.30 | 6.92 to 9.67 | Random | 24 | Low | IV |
| Peng et al [26] | 2022 | Arthroplasty | TUG | 3 | SMD | -0.34 | -1.31 to 0.63 | Random | 95 | Very Low | NS |
| Huang et al [27] | 2022 | Pain Management | Pain intensity | 28 | WMD | -1.62 | -1.86 to -1.38 | Fixed | 27 | High | III |
| Gazendam et al [28] | 2022 | Arthroplasty | VAS | 3 | MD | -3.30 | -8.03 to 1.43 | Random | 84 | Very Low | NS |
| Bordeleau et al [29] | 2022 | Back Pain | Pain intensity | 16 | SMD | -0.67 | -1.12 to -0.23 | Random | 85 | Moderate | IV |
| Cortés-Pérez et al [30] | 2021 | Fibromyalgia Syndrome in Women | VAS | 6 | SMD | -0.45 | -0.692 to -0.205 | NA | NA | Moderate | IV |
| Cortés-Pérez et al [30] | 2021 | Fibromyalgia Syndrome in Women | Dynamic balance | 3 | SMD | -0.76 | -1.12 to -0.392 | NA | NA | Low | IV |
| Cortés-Pérez et al [30] | 2021 | Fibromyalgia Syndrome in Women | Fatigue | 4 | SMD | -0.58 | -1.019 to -0.139 | NA | NA | Very Low | IV |
| Cortés-Pérez et al [30] | 2021 | Fibromyalgia Syndrome in Women | Quality of life | 5 | SMD | 0.55 | 0.299 to 0.810 | NA | NA | Low | IV |
| Cortés-Pérez et al [30] | 2021 | Fibromyalgia Syndrome in Women | Anxiety | 3 | SMD | -0.47 | -0.908 to -0.029 | NA | NA | Very Low | IV |
| Cortés-Pérez et al [30] | 2021 | Fibromyalgia Syndrome in Women | Depression | 4 | SMD | -0.46 | -0.764 to -0.158 | NA | NA | Low | IV |
| Wang et al [31] | 2019 | Arthroplasty | VAS | 3 | MD | -0.19 | -0.36 to -0.03 | Random | 0 | Low | IV |
| Wang et al [31] | 2019 | Arthroplasty | Six-minute walk test | 2 | MD | 29.36 | -6.99 to 65.71 | Random | 88 | Very Low | NS |
| Gumaa and Rehan Youssef [32] | 2019 | Orthopedic Rehabilitation | VAS | 3 | SMD | -0.24 | -0.61 to 0.12 | Random | 0 | Very Low | NS |
