# Supplementary material for: The Efficacy of Virtual Reality on the Rehabilitation of Musculoskeletal Diseases: Umbrella Review
Source: J Med Internet Res. 2025 Apr 25;27:e64576. doi: 10.2196/64576 (PMC12064964; doi:10.2196/64576)
Supplement: Multimedia Appendix 5 [file jmir_v27i1e64576_app5.docx]

| [1] | Inappropriate type of research |
| --- | --- |
| [2] | Inappropriate type of research |
| [3] | Inappropriate type of research |
| [4] | Inappropriate type of research |
| [5] | Inappropriate type of research |
| [6] | Inappropriate type of research |
| [7] | Inappropriate type of research |
| [8] | Inappropriate type of research |
| [9] | Inappropriate type of research |
| [10] | Inappropriate type of research |
| [11] | Inappropriate type of research |
| [12] | Inappropriate type of research |
| [13] | Inappropriate type of research |
| [14] | Inappropriate type of research |
| [15] | Inappropriate type of research |
| [16] | Inappropriate type of research |
| [17] | Inappropriate type of research |
| [18] | Inappropriate type of research |
| [19] | Inappropriate research topic |

Reference:

1. Su S, Wang R, Zhou R, Chen Z, Zhou F. The effectiveness of virtual reality, augmented reality, and mixed reality training in total hip arthroplasty: a systematic review and meta-analysis. Journal of orthopaedic surgery and research 2023;**18**(1):121 doi: 10.1186/s13018-023-03604-z.

2. Opara M, Kozinc Ž. Virtual reality training for management of chronic neck pain: a systematic review with meta-analysis. European Journal of Physiotherapy 2023 doi: 10.1080/21679169.2023.2215831.

3. Choi T, Heo S, Choi W, Lee S. A Systematic Review and Meta-Analysis of the Effectiveness of Virtual Reality-Based Rehabilitation Therapy on Reducing the Degree of Pain Experienced by Individuals with Low Back Pain. International Journal of Environmental Research and Public Health 2023;**20**(4) doi: 10.3390/ijerph20043502.

4. Grassini S. Virtual Reality Assisted Non-Pharmacological Treatments in Chronic Pain Management: A Systematic Review and Quantitative Meta-Analysis. International Journal of Environmental Research and Public Health 2022;**19**(7) doi: 10.3390/ijerph19074071.

5. Brea-Gómez B, Torres-Sánchez I, Ortiz-Rubio A, et al. Virtual reality in the treatment of adults with chronic low back pain: A systematic review and meta-analysis of randomized clinical trials. International Journal of Environmental Research and Public Health 2021;**18**(22) doi: 10.3390/ijerph182211806.

6. Ahern MM, Dean LV, Stoddard CC, et al. The Effectiveness of Virtual Reality in Patients With Spinal Pain: A Systematic Review and Meta-Analysis. Pain Practice 2020;**20**(6):656-75 doi: 10.1111/papr.12885.

7. Sun P, Zhao Y, Men J, et al. Application of Virtual and Augmented Reality Technology in Hip Surgery: Systematic Review. Journal of Medical Internet Research 2023;**25** doi: 10.2196/37599.

8. Kiani S, Rezaei I, Abasi S, Zakerabasali S, Yazdani A. Technical aspects of virtual augmented reality-based rehabilitation systems for musculoskeletal disorders of the lower limbs: a systematic review. BMC Musculoskelet Disord 2023;**24**(1):4 doi: 10.1186/s12891-022-06062-6 [published Online First: 20230103].

9. Dubron K, Verbist M, Jacobs R, Olszewski R, Shaheen E, Willaert R. Augmented and Virtual Reality for Preoperative Trauma Planning, Focusing on Orbital Reconstructions: A Systematic Review. Journal of Clinical Medicine 2023;**12**(16) doi: 10.3390/jcm12165203.

10. Sumdani H, Aguilar-Salinas P, Avila MJ, Barber SR, Dumont T. Utility of Augmented Reality and Virtual Reality in Spine Surgery: A Systematic Review of the Literature. World Neurosurgery 2022;**161**:e8-e17 doi: 10.1016/j.wneu.2021.08.002.

11. Matthie NS, Giordano NA, Jenerette CM, et al. Use and efficacy of virtual, augmented, or mixed reality technology for chronic pain: a systematic review. Pain Management 2022;**12**(7):859-78 doi: 10.2217/pmt-2022-0030.

12. Lin H, Han K, Ruan B. Effect of Virtual Reality on Functional Ankle Instability Rehabilitation: A Systematic Review. J Healthc Eng 2021;**2021**:7363403 doi: 10.1155/2021/7363403 [published Online First: 20211129].

13. Gumaa M, Khaireldin A, Rehan Youssef A. Validity and Reliability of Interactive Virtual Reality in Assessing the Musculoskeletal System: a Systematic Review. Current Reviews in Musculoskeletal Medicine 2021;**14**(2):130-44 doi: 10.1007/s12178-021-09696-6.

14. Pereira MF, Prahm C, Kolbenschlag J, Oliveira E, Rodrigues NF. Application of AR and VR in hand rehabilitation: A systematic review. Journal of Biomedical Informatics 2020;**111** doi: 10.1016/j.jbi.2020.103584.

15. Baeza-Barragán MR, Labajos Manzanares MT, Ruiz Vergara C, Casuso-Holgado MJ, Martín-Valero R. The Use of Virtual Reality Technologies in the Treatment of Duchenne Muscular Dystrophy: Systematic Review. JMIR Mhealth Uhealth 2020;**8**(12):e21576 doi: 10.2196/21576 [published Online First: 20201208].

16. De Araújo AVL, Neiva JFDO, Monteiro CBDM, Magalhães FH. Efficacy of Virtual Reality Rehabilitation after Spinal Cord Injury: A Systematic Review. BioMed Research International 2019;**2019** doi: 10.1155/2019/7106951.

17. Dascal J, Reid M, Ishak WW, et al. Virtual reality and medical inpatients: A systematic review of randomized, controlled trials. Innovations in Clinical Neuroscience 2017;**14**(1-2):14-21.

18. Miller KJ, Adair BS, Pearce AJ, Said CM, Ozanne E, Morris MM. Effectiveness and feasibility of virtual reality and gaming system use at home by older adults for enabling physical activity to improve health-related domains: A systematic review. Age and Ageing 2014;**43**(2):188-95 doi: 10.1093/ageing/aft194.

19. Li Y, Huang J, Li X, et al. Effect of Time-Dose-Matched Virtual Reality Therapy on Upper Limb Dysfunction in Patients Poststroke: A Meta-Analysis of Randomized Controlled Trials. Archives of Physical Medicine and Rehabilitation 2022;**103**(6):1131-43.e7 doi: 10.1016/j.apmr.2021.09.003.
