# Supplementary material for: The Efficacy of Virtual Reality on the Rehabilitation of Musculoskeletal Diseases: Umbrella Review
Source: J Med Internet Res. 2025 Apr 25;27:e64576. doi: 10.2196/64576 (PMC12064964; doi:10.2196/64576)
Supplement: Multimedia Appendix 6 [file jmir_v27i1e64576_app6.docx]

| Study | Year | Key findings |
| --- | --- | --- |
| Su et al [19] | 2024 | Based on the pooled results, we suggested that VR-based rehabilitation may have benefit in patients’ postoperative rehabilitation in TKA. |
| Li et al [20] | 2024 | These findings indicated that virtual reality–based training can be used effectively for individuals with chronic low back pain in the immediate term, especially to reduce pain, alleviate pain-related fear, and improve disability. |
| Hao et al [21] | 2024 | Virtual reality is a promising intervention to address disability and pain in patients with chronic neck pain. |
| Guo et al [22] | 2024 | The meta-analysis review conducted in this study revealed that VR intervention may potentially improve balance among the patients with knee joint pain. |
| Ye et al [23] | 2023 | VR interventions demonstrated significant improvement in patients experiencing Neck Pain. |
| Kantha et al [24] | 2023 | VR is recommended for reducing pain intensity more than no rehabilitation or conventional rehabilitation. |
| Guo et al [25] | 2023 | Existing moderate evidence support VR as a beneficial nonpharmacological approach to improve pain intensity in patients with neck pain. |
| Peng et al [26] | 2022 | VR-based rehabilitation improved pain and function but not postural control following TKA compared to conventional rehabilitation. |
| Huang et al [27] | 2022 | VR is a feasible alternative therapy for both juveniles and adults in pain management, and it has a greater potential for juveniles |
| Gazendam et al [28] | 2022 | VR-based rehabilitation for patients undergoing TKA represents an evolving field that may have advantages  over traditional therapy for some patients. |
| Bordeleau et al [29] | 2022 | Extended reality technologies have appeared as interesting nonpharmacological options for the treatment of back pain, with the potential to minimise the need for opioid medications. |
| Cortés-Pérez et al [30] | 2021 | VR is an effective therapy that reduces the impact of FMS, pain, fatigue, anxiety and depression and increases dynamic balance, aerobic capacity and quality of life in women with FMS. |
| Wang et al [31] | 2019 | There is moderate-quality of evidence showed technology-assisted rehabilitation, in particular, telerehabilitation, results in a statistically significant improvement in pain. |
| Gumaa and Rehan Youssef [32] | 2019 | The evidence of VR effectiveness is promising in chronic neck pain and shoulder impingement syndrome. |
